# Upstream CtrA-binding sites both induce and repress pilin gene expression in *Caulobacter crescentus*

**DOI:** 10.1186/s12864-024-10533-6

**Published:** 2024-07-19

**Authors:** Anurag Rijal, Eli T. Johnson, Patrick D. Curtis

**Affiliations:** Department of Biology, University of Mississippi, University, 402 Shoemaker Hall, Oxford, MS 38677 USA

**Keywords:** CtrA, *pilA*, *Caulobacter crescentus*, Transcriptional regulation

## Abstract

Pili are bacterial surface structures important for surface adhesion. In the alphaproteobacterium *Caulobacter crescentus*, the global regulator CtrA activates transcription of roughly 100 genes, including *pilA* which codes for the pilin monomer that makes up the pilus filament. While most CtrA-activated promoters have a single CtrA-binding site at the − 35 position and are induced at the early to mid-predivisional cell stage, the *pilA* promoter has 3 additional upstream CtrA-binding sites and it is induced at the late predivisional cell stage. Reporter constructs where these additional sites were disrupted by deletion or mutation led to increased activity compared to the WT promoter. In synchronized cultures, these mutations caused *pilA* transcription to occur approximately 20 min earlier than WT. The results suggested that the site overlapping the − 35 position drives *pilA* gene expression while the other upstream CtrA-binding sites serve to reduce and delay expression. EMSA experiments showed that the − 35 Site has lower affinity for CtrA∼P compared to the other sites, suggesting binding site affinity may be involved in the delay mechanism. Mutating the upstream inhibitory CtrA-binding sites in the *pilA* promoter caused significantly higher numbers of pre-divisional cells to express pili, and phage survival assays showed this strain to be significantly more sensitive to pilitropic phage. These results suggest that *pilA* regulation evolved in *C. crescentus* to provide an ecological advantage within the context of phage infection.

**Supplementary Information:**

The online version contains supplementary material available at 10.1186/s12864-024-10533-6.

## Introduction

Surface attachment provides several advantages for the bacterial cell, including mediating host pathogen interactions [1, 2], biofilm formation [3], and cell motility [4, 5]. An important appendage that aids bacteria in surface attachment is the pilus, composed of several identical 15–40 kDa protein subunits called pilins that are polymerized into a linear filament [4]. The freshwater alphaproteobacterium *Caulobacter crescentus* produces pili belonging to a specific subtype of the Type IVb pilus family, called the Tad/Cpa pilus system [6, 7]. Pilins of this system are smaller (50–80 aa) than other mature Type IVb pili (180–200 aa), and the 4 core minor pilins present in all other Type IVb pili systems are absent in these systems [8, 9]. Moreover, *C. crecentus* Tad pili have been found to retract despite lacking the known ATPases responsible for pilus retraction found in Type IV pilus systems [10]. Other than facilitating surface attachment and motility, Tad pili in *C. crescentus* have also been reported to stimulate surface sensing deployment of the adhesive polysaccharide holdfast and surface colonization [10, 11]. In *C. crescentus*, pili are formed at a specific stage of the life cycle. *C. crescentus* has a complex dimorphic lifestyle where a predivisional mother cell divides asymmetrically to give rise to a replication-deficient motile swarmer cell with pili and a flagellum at the same pole, and a replication-capable sessile cell that contains an extension of the cell envelope at one pole called the stalk. The swarmer cell eventually sheds the flagellum and retracts the pili to initiate differentiation into a stalked cell, thereby completing the cell cycle.

Cell cycle progression in *C. crescentus* is primarily regulated by three global regulators DnaA, GcrA and CtrA, all of which reach peak abundance at different times in the cell cycle [12]. Pilus biogenesis is coordinated by these regulators. Synthesis of the basal bodies of the pilus is induced by GcrA and become fully assembled before the end of the predivisional cell stage [7, 13–15], but the prepilin peptidase CpaA and the pilus filament monomer PilA are expressed by CtrA and the assembly of the pilus filaments happens only in swarmer cells [14, 16]. During the swarmer cell stage, pili retract giving rise to an intracellular pool of pilin subunits which are then recycled and reused in newly extended pilus filaments [10]. The disappearance of PilA protein during the swarmer to stalked cell transition suggests the pilin monomers are degraded, though this has not been directly shown. CtrA, which has been dubbed the master regulator of the *C. crescentus* cell cycle, directly controls the expression of about 95 genes in *C. crescentus*, and is known to affect the transcription of nearly 25% of all cell cycle regulated genes directly or indirectly [17]. CtrA is a response regulator functioning in a phosphorelay with the hybrid histidine kinase CckA and Hpt protein ChpT [18–20]. In addition to proteolysis and phosphorylation, regulation of CtrA activity is also controlled by timing of its synthesis. CtrA synthesis is induced by GcrA starting during the early predivisional cell stage and peaks during the late-predivisional and swarmer cell stages [21]. CtrA-dependent gene expression typically matches the activity profile of CtrA [16, 17]. However, *pilA* transcripts reach peak abundance only when cell compartmentalization has completed [7], later than most CtrA-dependent genes. Therefore, it is evident that *pilA* regulation has additional factors.

**Fig. 1:**
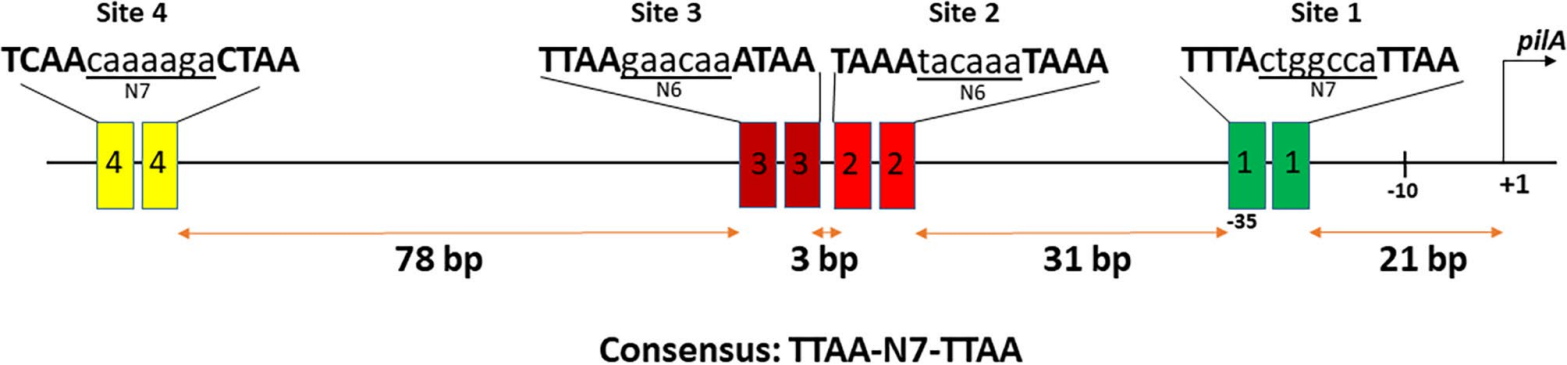
The *pilA* promoter architecture in *C. crescentus* with four CtrA-binding sites upstream of transcription start site. Site 1 (green) overlaps the − 35 position, Site 2 (red) is located 31 bp upstream of Site 1, Site 3 (maroon) is located 3 bp upstream of Site 2 and Site 4 (yellow) is positioned 78 bp upstream of Site 3. The nucleotide sequence for each binding site is provided between the extended lines with uppercase letters representing the half-sites. Site 1 is closest to the consensus CtrA-binding site TTAA-N7-TTAA with a single nucleotide deviation. The nucleotide sequences of the CtrA-binding sites in the *pilA* promoter was derived from [14].

There are several unusual aspects in the *pilA* promoter architecture. First, most CtrA-induced promoters have a single near consensus CtrA-binding motif at the − 35 position. The *pilA* promoter, however, has four CtrA binding sites as determined by footprinting [14]. One site overlaps the − 35 position with additional sites found upstream (diagrammed in Fig. 1). Second, all these sites have varying degrees of deviation from the consensus CtrA-binding motif TTAA-N7-TTAA, with Site 1 being the closest to consensus and the additional sites more divergent. The spacing between sites is inconsistent; Site 2 is positioned 31 bases upstream of Site 1, Sites 2 and 3 are just 3 bases apart, while Site 4 is located 78 bases upstream of Site 3 (Fig. 1). Thirdly, compared to several other CtrA-dependent promoters, the *pilA* promoter is more sensitive to changes in intracellular CtrA levels. A mutation that drastically reduced CtrA activity had little impact on promoters with single CtrA binding sites, but the *pilA* promoter showed a significant decrease in activity [22].

All these facts put together suggest a model where the additional upstream CtrA-binding sites contribute to the unique timing and sensitivity of *pilA* promoter. This study examines this hypothesis through mutational, physiological, and biochemical analyses. The results support the hypothesis that these upstream sites serve to delay transcriptional activity of the *pilA* promoter, and suggest that binding affinity may be important to the underlying mechanism. Moreover, the results suggest an ecological advantage for this delay, providing a clear motivation for the evolution of this mechanism.

## Results

### Upstream CtrA-binding sites repress *pilA* transcription

**Fig. 2:**
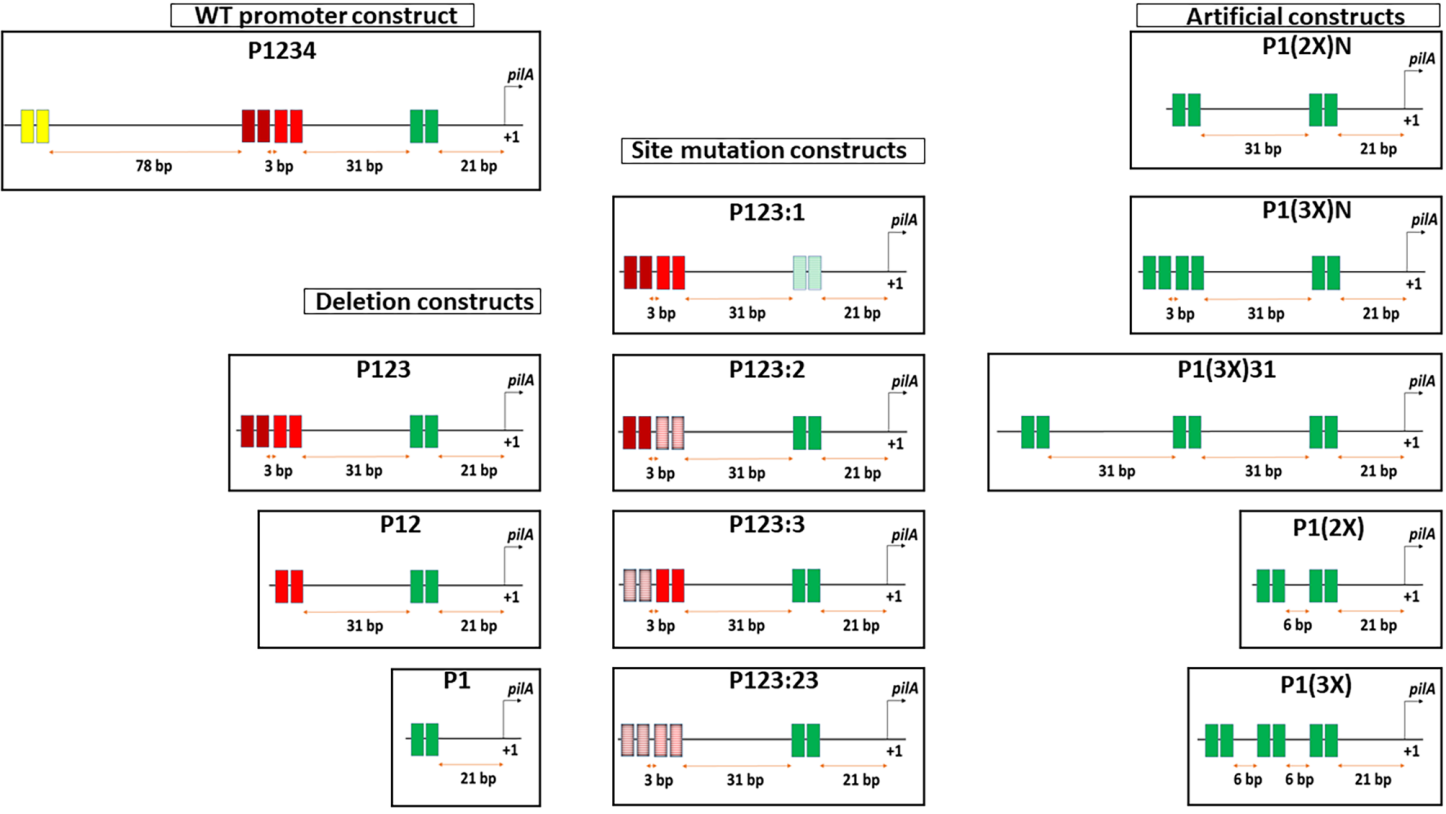
Schematic representation of the P_*pilA*_ architecture in the intact and mutated/artificial P_*pilA*_-*lacZ* constructs used for β-galactosidase assay. Each rectangle indicates a single half-site. Site 1 nucleotide sequence is indicated in green, Site 2 in red, Site 3 in maroon and Site 4 in yellow. The numbers below the arrows indicate the spacing between the CtrA-binding sites. Mutated sites are indicated by a stripes inside the respective rectangles representing the half-sites.

**Fig. 3:**
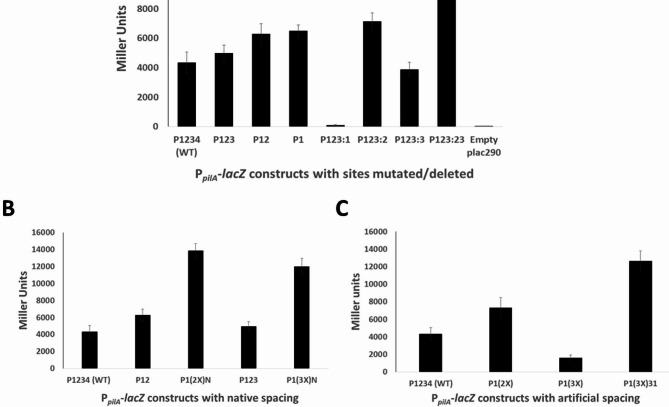
Sites 2, 3 and 4 repress P_*pilA*_ activity, while Site 1 drives expression. β-galactosidase assays were done for various P_*pilA*_-*lacZ* constructs in *C. crescentus* NA1000 wild-type strain. A: Promoter activity for constructs with native, truncated, and mutated CtrA-binding sites. Other than the WT construct P1234, all constructs have Site 4 deleted. P123 has Site 4 deleted; P12 has Sites 3 and 4 deleted; P1 has Sites 2, 3 and 4 deleted; P123:1 has Site 1 mutated; P123:2 has Site 2 mutated; P123:3 has Site 3 mutated and P123:23 has Sites 2 and 3 mutated. Mutated sites had each TTAA half-site replaced by GGCC. The empty plac290 construct with a promoterless *lacZ* gene is the negative control. B: Comparison of promoter activity of wild-type construct with constructs where nucleotide sequence of Site 1 is used to replace the native sequence of Sites 2 and 3. P1(2X)N has the second copy of Site 1 at Site 2’s native position with other sites deleted. P1(3X)N has Sites 2 and 3 replaced by Site 1 sequence at native spacing. C: Comparison of promoter activity of wild-type with constructs having artificial spacing (6 bp) between duplicate and triplicate copies of Site 1. P1(2X) has second Site 1 copy 6 bp upstream of native Site 1 with other sites deleted. P1(3X) is similar to P1(2X) except that it has a third Site 1 copy 6 bp upstream of the second copy. P1(3X )31 has two additional Site 1 copies each 31 bp upstream of the previous copy.

The fact that *pilA* is expressed later in the cell cycle compared with other CtrA-dependent genes, combined with the unusual promoter of *pilA* that contains multiple CtrA binding sites, suggests that these additional sites may play a role in *pilA* expression. To assess if these additional sites impact *pilA* expression, the role of each individual CtrA-binding site in the *pilA* promoter was determined by creating transcriptional fusions of intact as well as mutated versions of the promoter to a promoterless *lacZ* gene in a low-copy number plasmid (plac290) and performing β-galactosidase assays in the wild-type *C. crescentus* NA1000 strain. Mutated promoters were created by either truncating the promoter to progressively remove distal binding sites, or mutating both half-sites in a binding site to GGCC, which has been previously reported to prevent CtrA binding [23]. A schematic representation of all *pilA* promoter constructs created for this assay is shown in Fig. 2. As shown in Fig. 3A, Site 1 by itself (P1) was sufficient to drive expression, which is expected given that it overlaps the − 35 region of the promoter, which has been shown to induce expression in other CtrA-dependent promoters [24, 25]. In concordance with this result, mutation of Site 1 (P123:1) abolished expression. The initially surprising result was that deleting Sites 2–4 collectively (P1) increased expression compared to the WT promoter. This result suggests that these additional binding sites repress expression from upstream positions. Deletion of Site 4 (P123) did not substantially alter expression, suggesting this most distal site had little impact on *pilA* expression. Thus, Site 4 was excluded from all other constructs. While individually mutating Site 3 (P123:3) had little impact on expression, mutating Site 2 (P123:2) increased activity to nearly twice the activity of the wild-type (P1234). Interestingly, P123:23 with Site 4 deleted and Sites 2 and 3 mutated had the highest activity among the native promoter constructs, despite Site 3 on its own not appearing to provide any repression, suggesting potential cooperativity in their repression activity. Furthermore, the construct P12 which had the same composition in terms of active CtrA-binding sites as P123:3 and was expected to repress expression because of the presence of a functional Site 2, instead had the same activity as P1. Either Site 3 is necessary for Site 2 to inhibit expression, or the nucleotides around and within the Site 3 position, not necessarily its CtrA-binding nucleotide sequence, function with Site 2 to exert its repressive effect.

In order to further dissect the importance of the nucleotide sequence of Sites 2 and 3, sequences of Sites 2 and 3 were replaced at their native positions by copies of Site 1. P1(2X)N had intact Site 1 at its native position and another copy of Site 1 replacing the nucleotide sequence of Site 2 at its native position, with other sites deleted. P1(3X)N was similar to P1(2X)N, except that it had a third copy of Site 1 replacing the nucleotide sequence of Site 3 at its native position, with Site 4 deleted. Both P1(2X)N and P1(3X)N had significantly higher activity than the WT construct and the corresponding deletion constructs (P12 and P123 respectively, Fig. 3B). The results suggest that the specific sequences of Sites 2 and 3 contribute to their inhibitory effect. Both Sites 2 and 3 deviate from the CtrA consensus binding motif, not only in the individual bases, but they also have a 6 bp spacing between the half-sites instead of the consensus 7 bp spacing (Figs. 1 and 2).

Next, to determine how the spacing between the Site 1 copies would affect activity, promoter constructs with tandem Site 1 copies separated by 6 bp were created. The 6 bp spacing was chosen so that it was similar to the 6–7 bp spacing found between half-sites of the CtrA-binding sites in the *pilA* promoter. The P1(2X) construct with a second copy of Site 1 placed 6 bp upstream of the native copy and all other CtrA-binding sites deleted showed similar activity to P1 and P12, whereas P1(3X) with a third copy of Site 1 6 bp upstream of the second copy showed the lowest activity, amounting to less than half the activity of the wild-type construct P1234 (Fig. 3C). This was contrary to the P1(3X)N results which also has three Site 1 copies, just at different positions. On the other hand, a construct with three Site 1 copies separated by 31 bp instead of 6 bp [P1(3X )31] resulted in activities similar to P1(3X)N. These results suggest that not only are the sequences of the additional upstream sites important for repression, but that the relative spacing of upstream sites with respect to Site 1 are also important for upstream repression.

### Sites 2 and 3 increase the *pilA* promoter’s sensitivity to CtrA levels

**Fig. 4:**
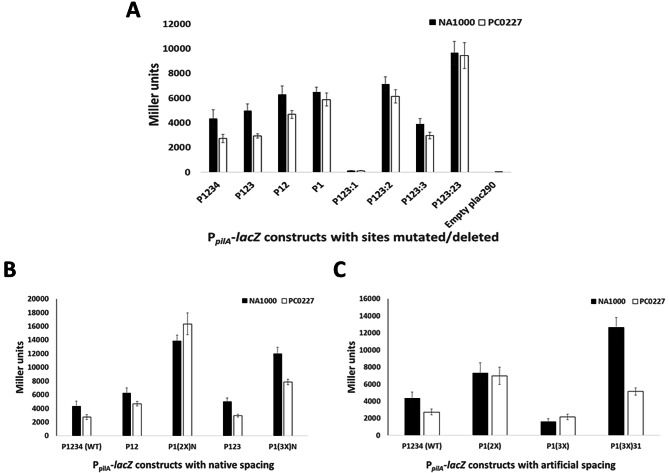
Sites 2 and 3 increase P_*pilA*_ sensitivity to CtrA levels. β-galactosidase assays were compared for the various P_*pilA*_-*lacZ* constructs between *C. crescentus* NA1000 wild-type strain and the low-CtrA abundance strain PC0227. Strain PC0227 contains a transposon insertion into the *ctrA* P2 promoter that causes a reduction in CtrA abundance, derived from strain YB3558. A: Promoter activity for constructs with native, truncated and mutated CtrA-binding sites. Other than the WT construct P1234, all constructs have Site 4 deleted. P123 has Site 4 deleted; P12 has Sites 3 and 4 deleted; P1 has Sites 2, 3 and 4 deleted; P123:1 has Site 1 mutated; P123:2 has Site 2 mutated; P123:3 has Site 3 mutated and P123:23 has Sites 2 and 3 mutated. Mutated sites had each TTAA half-site replaced by GGCC. The empty plac290 construct with a promoterless *lacZ* gene is the negative control. Similarly, the P_*xyl*_-*lacZ* construct is a negative control for CtrA-dependent transcription, which shows no change in activity between WT and PC0227. B: Promoter activity for constructs where nucleotide sequence of Site 1 is used to replace the native sequence of Sites 2 and 3. P1(2X)N has the second copy of Site 1 at Site 2’s native position with other sites deleted. P1(3X)N has Sites 2 and 3 replaced by Site 1 sequence at native spacing. C: Promoter activity of constructs having artificial spacing (6 bp) between duplicate and triplicate copies of Site 1. P1(2X) has second Site 1 copy 6 bp upstream of native Site 1 with other sites deleted. P1(3X) is similar to P1(2X) except that it has a third Site 1 copy 6 bp upstream of the second copy. P1(3X  )31 has two additional Site 1 copies each 31 bp upstream of the previous copy.

It had been previously shown that a transposon insertion into the strong P2 promoter of *ctrA* causes CtrA levels to be reduced to roughly 25% of the wild-type (strain YB3558), and that this mutation caused a significant reduction in *pilA* expression but had little impact on most other CtrA-dependent promoters [22]. Interestingly, among the few promoters that were at least moderately affected, the *pilA* promoter was the only one which had multiple CtrA-binding sites. Thus, it was hypothesized that the presence of these additional binding sites contribute to this phenomenon and to investigate this, β-galactosidase assays were performed for intact and mutant P_*pilA*_-*lacZ* constructs in WT and PC0227 (where the YB3558 mutation has been transduced from the CB15 background to the NA1000 background). The PC0227 strain, similar to YB3558, has morphological and growth deficiencies, as evidenced by its filamentation phenotype and slower growth rate with a doubling time of 129 min compared to the 105 min for the WT (Supplementary Figure S2). The activity of the native *pilA* promoter in this low-CtrA strain reduced to about 60% of that of the wild-type strain indicating that the native *pilA* promoter with all four binding sites intact is sensitive to changes in CtrA levels (Fig. 4A). A similar level of reduction was seen for P123 with Site 4 deleted, which suggests that Site 4 does not play a role in this sensitivity of P_*pilA*_ to CtrA levels. P12 with Sites 3 and 4 deleted in PC0227 also showed reduced activity, but the reduction was less severe than that of P1234 (75% of expression in the WT strain). This gradual reduction in sensitivity continued for P1 in PC0227 with 90% activity of that in WT. A similar pattern was observed for the mutation constructs where P123:2 and P123:3 showed 86% and 76% activity in PC0227 compared to the WT, whereas the sensitivity was completely abolished in P123:23 where both Sites 2 and 3 were mutated. Thus, there was a clear pattern observed where individual mutations/deletions of Sites 2 or 3 reduced sensitivity of P_*pilA*_ to CtrA levels, with the reduction being more drastic with Site 2 mutation compared to Site 3 mutation and mutation of both sites resulted in virtually no difference in the activities between the WT and the PC0227 strain. Furthermore, activity for P1(2X)N was slightly higher in PC0227 than the WT strain, whereas that of P1(3X)N was about 65% of that in the WT strain (Fig. 4B), indicating that the presence of 3 sites at their native positions was sufficient for increasing the promoter’s sensitivity to CtrA levels, irrespective of the nucleotide sequence. In the case of the constructs with artificial spacing (Fig. 4C), there was no difference in activity between WT and PC0227 for P1(2X) and P1(3X), but activity of P1(3X) 31 in PC0227 was reduced to about 40% of that in the WT strain, suggesting that increasing the spacing between binding sites results in increased sensitivity to CtrA levels.

### Sites 2 and 3 collectively play a role in delaying transcription from the *pilA* promoter

**Fig. 5:**
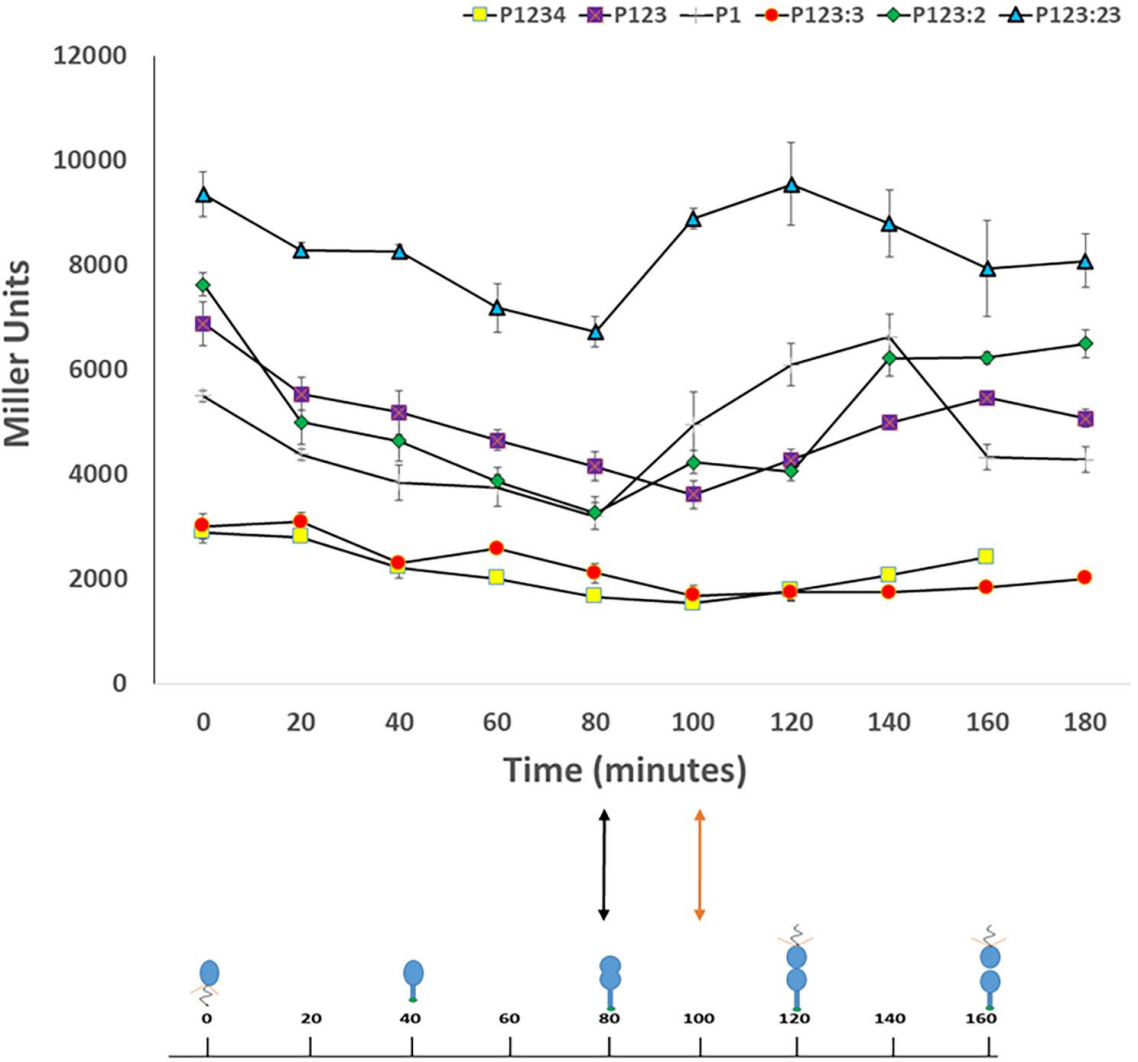
Sites 2 and 3 delay P_*pilA*_ expression. Gene expression of WT and mutated *pilA* promoter constructs were measured by β-galactosidase assay every 20 min during the course of the cell cycle. Generally, transcription was found to decrease initially, then increase in the predivisional cell stage. Transcription was found to initiate 20 min earlier for constructs P1, P123:2 and P123:23 at the early pre-divisional stage (indicated by black double-headed arrow) as compared to constructs P1234 (WT), P123 and P123:3 (indicated by orange double-headed arrow).

The results thus far suggest that Sites 2 and 3 repress *pilA* transcription, but these results were obtained from bulk cultures where cells are in various stages of the cell cycle and do not address the timing aspect of *pilA* expression. In order to determine the effects Sites 2 and 3 have on the timing of *pilA* transcription, *C. crescentus* strains with the various P_*pilA*_-*lacZ* constructs were synchronized and β-galactosidase activity was measured at regular time intervals. All strains showed activity at the start of the synchrony which decreased for the first few timepoints, suggesting either residual *pilA* expression during the swarmer cell stage or persistence of the β-galactosidase enzyme through this stage (Fig. 5). A modest increase in activity was observed for strains with constructs P1234, P123 and P123:3 between 100 and 120 min, corresponding to the mid-to-late predivisional cell stages. This timing is consistent with the published timing of *pilA* expression during the cell cycle [14]. Conversely, a sharp increase in promoter activity was observed for strains with constructs P1, P123:2 and P123:23 between 80 and 100 min of the cell cycle, corresponding to the early to mid-predivisional cell stages and roughly 20 min earlier expression of *pilA*. These are the same constructs that show increased expression in bulk culture, suggesting that Sites 2 and 3 not only play a role in repressing *pilA* transcription, but also delay *pilA* transcription until the mid-to-late predivisional cell stage. The role of Site 3 individually is unclear as the individual Site 3 mutation (P123:3) resulted in little change to expression timing, while Site 2 mutation (P123:2) led to earlier and stronger expression, but mutation of both (P123:23) led to even stronger expression than Site 2 mutated alone and at the same earlier time point. Perhaps Site 3 amplifies the inhibitory effect of Site 2.

### Mutating sites 2 and 3 in the *pilA* promoter causes earlier and greater PilA accumulation

**Fig. 6:**
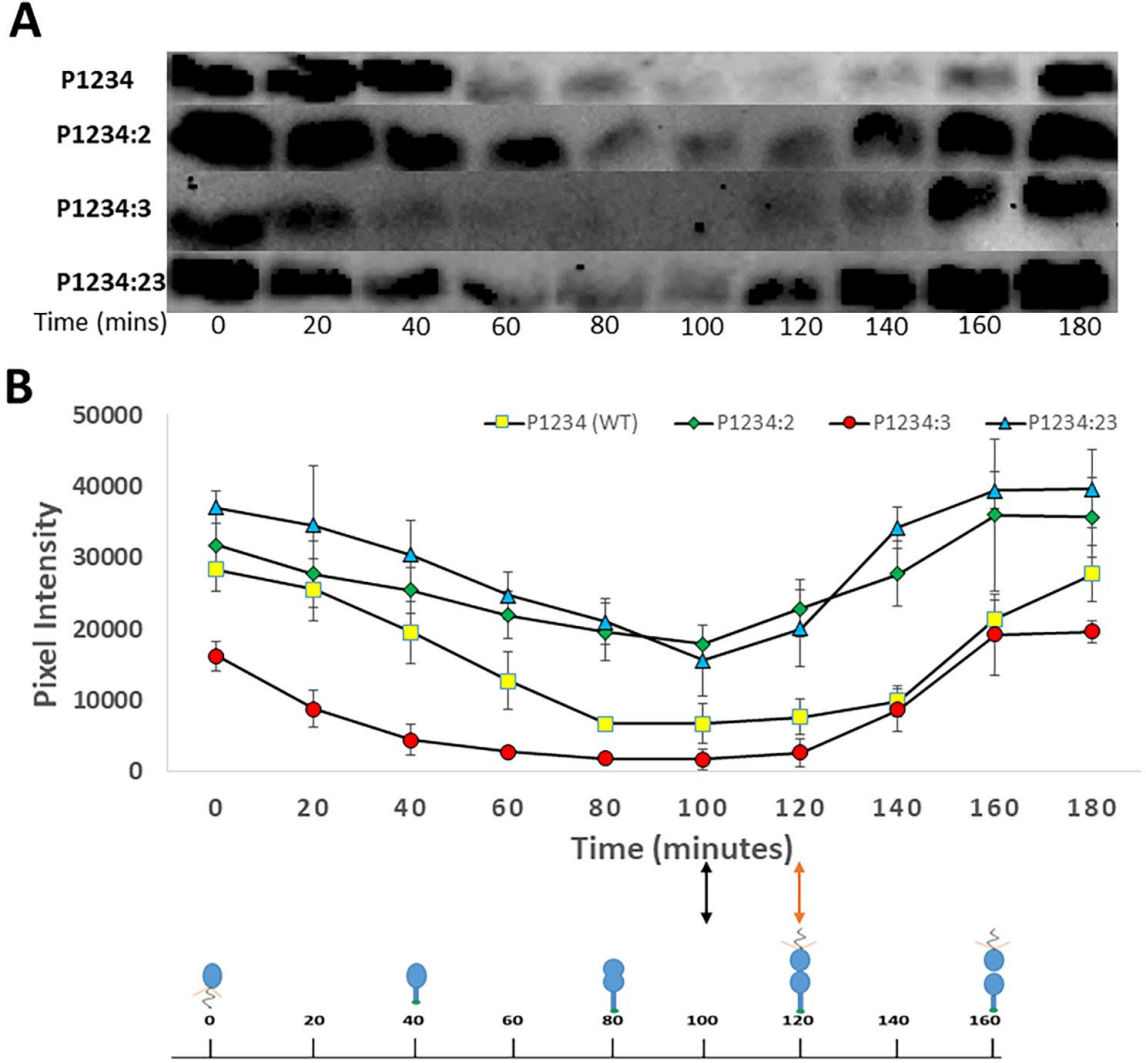
PilA accumulation occurs earlier and in greater quantity in P1234:23. PilA production was assessed over the course of the cell cycle for WT and mutated *pilA* promoters by anti-PilA western blots. Western blots were performed with uniform protein concentrations, antibody concentration, and exposure time. A: Representative western blots with synchronized cultures of P1234, P1234:2, P1234:3 and P1234:23 showing PilA accumulation over different timepoints during the cell cycle. Uncropped western blots have been included in Supplementary Figures S18-S21. B: Line graph depicting average PilA band pixel intensity values over different timepoints during the cell cycle for the above mentioned strains. The values are averages from 3 western blots done for 3 separate synchronized cultures. Similar to the β-galactosidase data, PilA was found to accumulate approximately 20 min earlier and in greater quantities for P1234:2 and P1234:23 (indicated by black double-headed arrow) compared to the other strains (indicated by orange double-headed arrow).

The results presented in the previous section have two complications. First, the ﻿β-galactosidase enzyme likely has significant persistence in the cell, reducing the temporal resolution of the assay. Second, while there has not been any observation of post-transcriptional regulation of *pilA*, altered *pilA* expression may not necessarily lead to altered PilA production or altered pilus synthesis. In order to determine what effect altered *pilA* transcription has on the timing and amount of PilA accumulation in *C. crescentus*, western blots were performed with an anti-PilA antibody on synchronized cultures of strains with chromosomal mutations in CtrA-binding sites of the P_*pilA*_ promoter and compared with the wild type strain. Western blots were performed in triplicate and densitometry of bands were measured, normalized to WT, and average and standard deviation were calculated (Fig. 6). It should be noted that all strains in this assay contained a *pilA*^*T36C*^ mutation that introduces a surface exposed cysteine on the PilA protein that is used in later experiments (see below). This mutation does not interfere with gene expression nor does it interfere with PilA western blot detection [10]. A western blot was also done for P1234:1 (PC0467) using whole cell lysate from overnight culture and no PilA band was detected (data not shown), indicating that PilA does not accumulate in cells that have a Site 1 mutation, consistent with the results from the β-galactosidase assay. Similar to the β-galactosidase results, PilA was produced more and earlier for P1234:2 (PC0468) and P1234:23 (PC0414) strains as compared to the wild type and the P1234:3 (PC0465) strain (Fig. 6). These results provided further evidence to support the hypothesis that Site 2, to an extent together with Site 3, delays *pilA* transcription and thus PilA accumulation so that the pilus filament is only expressed at the very end of cell division.

### CtrA binding sites 2 and 3 increase affinity of CtrA for the *pilA* promoter

**Fig. 7:**
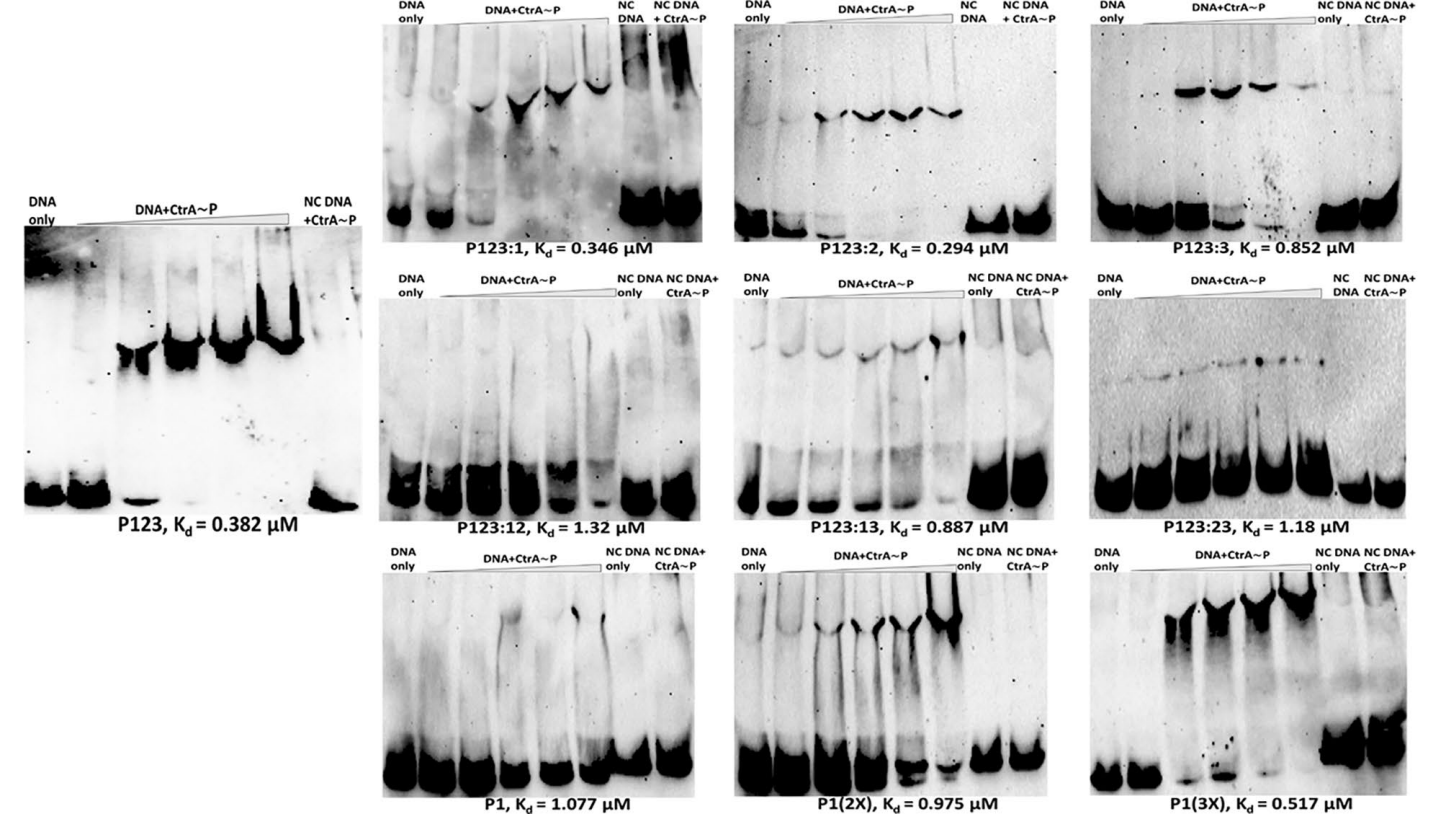
Sites 2 and 3 increase P_*pilA*_ affinity to CtrA. EMSA experiments were performed with WT and mutated promoter fragments to assess the binding affinity of phosphorylated CtrA. All lanes contained 100 ng DNA and 1 ng Poly didC. NC indicates a negative control DNA fragment that has all half-sites in Sites 1, 2 and 3 mutated to GGCC and Site 4 is absent. The first lane had DNA only and the second to sixth lanes had DNA and 0.17 µM, 0.33 µM, 0.5 µM, 0.66 µM and 1.65 µM of phosphorylated CtrA respectively. The last two lanes were controls with NC DNA only and NC DNA with 1.65 µM of phosphorylated CtrA respectively. Constructs with Sites 2 and/or 3 generally had more robust shifts, indicating these sites had higher affinity for phosphorylated CtrA. The dissociation constant (K_d_) for each construct has been listed at the bottom of the respective EMSA image. Gels have been cropped to remove background and uncropped gels have been included in Supplementary Figures S3-S12.

The results presented so far are consistent with CtrA-binding Sites 2 and (to a lesser extent) 3 repressing and delaying *pilA* expression, and Site 1 driving expression (though not necessarily at the same time), suggesting that CtrA performs a dual role at this promoter. To explore how these additional upstream binding sites impact CtrA binding dynamics, electrophoretic mobility shift assays (EMSAs) were performed with several different versions of the *pilA* promoter to see how CtrA∼P binding affinity changes. The P123 probe showed binding to CtrA∼P, with a probe shift seen at the second lowest CtrA∼P concentration and a slight supershift observed with increasing CtrA∼P concentration (K_d_ = 0.382 µM), though the oligomeric nature of the shift or supershift could not be determined (Fig. 7). Based on the results from β-galactosidase assays and the fact that the Site 1 sequence is the closest to the consensus CtrA-binding site, it was expected that Site 1 would have the highest affinity to CtrA∼P. However, the Site 1 only probe (P1) along with the P123:23 probe with Sites 2 and 3 mutated showed minimal shift (K_d_ = 1.077, 1.018 µM, respectively). This result was further supported by the fact that the Site 1 mutated probe (P123:1) still had the same binding profile (K_d_ = 0.346 µM) as the WT probe. Mutating Site 2 with Sites 1 and 3 intact (P123:2) still resulted in a shift (K_d_ = 0.294 µM) even with low CtrA∼P concentration, but there was no supershift as observed with P123 and P123:1. CtrA∼P bound to P123:3 with lower affinity (K_d_ = 0.852 µM) as evidenced by the thicker bottom bands in the first few lanes with CtrA∼P, although the pattern was largely the same as P123:2. These results indicate that intact sites 2 and 3 are required for highest affinity binding, with Site 3 appearing to be more important for high affinity binding than Site 2. However, mutating Sites 1 and 2 with Site 3 intact (P123:12) resulted in significantly reduced binding affinity (K_d_ = 1.32 µM), which decreased even further upon mutating Sites 1 and 3 and keeping Site 2 intact (P123:13) (K_d_ = 0.887 µM) indicating that the presence of intact Site 1 is necessary for high affinity binding when either Site 2 or 3 is mutated. Overall, Site 1 by itself (P1) or with Sites 2 and 3 mutated (P123:23) led to the highest *pilA* expression (Fig. 3A) and PilA protein levels (Fig. 6), but these constructs had the lowest CtrA∼P affinity (Fig. 7). In order to determine if the phosphorylation state of CtrA impacts binding, separate EMSAs were done with the P123 and P123:23 probes that had CtrA and CtrA∼P added to different lanes within the same gel (Supplementary Figures S13 and S14). The binding affinity of P123 for CtrA∼P was significantly higher than that for CtrA (K_d_ = 0.252, 0.745 µM respectively) while P123:23 showed little change between CtrA and CtrA∼P (K_d_ = 1.006, 1.085 µM respectively), suggesting that CtrA-phosphorylation does significantly increase its binding affinity to the WT *pilA* promoter. It should be noted that these assays had additional glycerol in the gel, so K_d_ values may not be directly comparable to those in Fig. 7.

EMSAs were also performed with the two artificial Site 1 duplication and triplication probes, P1(2X) and P1(3X) respectively. Having an extra copy of Site 1 6 bp upstream of the native copy in P1(2X) did result in increased binding affinity as compared to P1 (Fig. 7). Similarly, when a third copy was added in P1(3X), the affinity increased significantly with a supershift observed as in the case of P123. The P1 and P123:23 promoters have elevated expression compared to WT but with the lowest CtrA affinity. Here the P1(3X) probe has a much higher binding affinity compared to P1(2X) (Fig. 7), but had the largest reduction in gene expression (Fig. 3C), less than half of what was seen for the wild-type promoter. These results are consistent with an inverse relationship between CtrA affinity and gene expression. This result also suggests that the addition of the third CtrA site in this artificial promoter provided the majority of the inhibition. It is worth noting that this third site is 27 bp from the first site, and in the WT promoter, Site 2 (which provides the majority of the inhibition) is 31 bp from Site 1, suggesting that the spacing of sites is important for upstream inhibition. These results may suggest an alternative mechanism for regulation, where upstream sites are not sequestering CtrA from Site 1 but may oligomerize with CtrA at Site 1, dictated by spacing, in a way that is counterproductive to gene expression.

### Mutation of sites 2 and 3 induces earlier pilus filament production

**Fig. 8:**
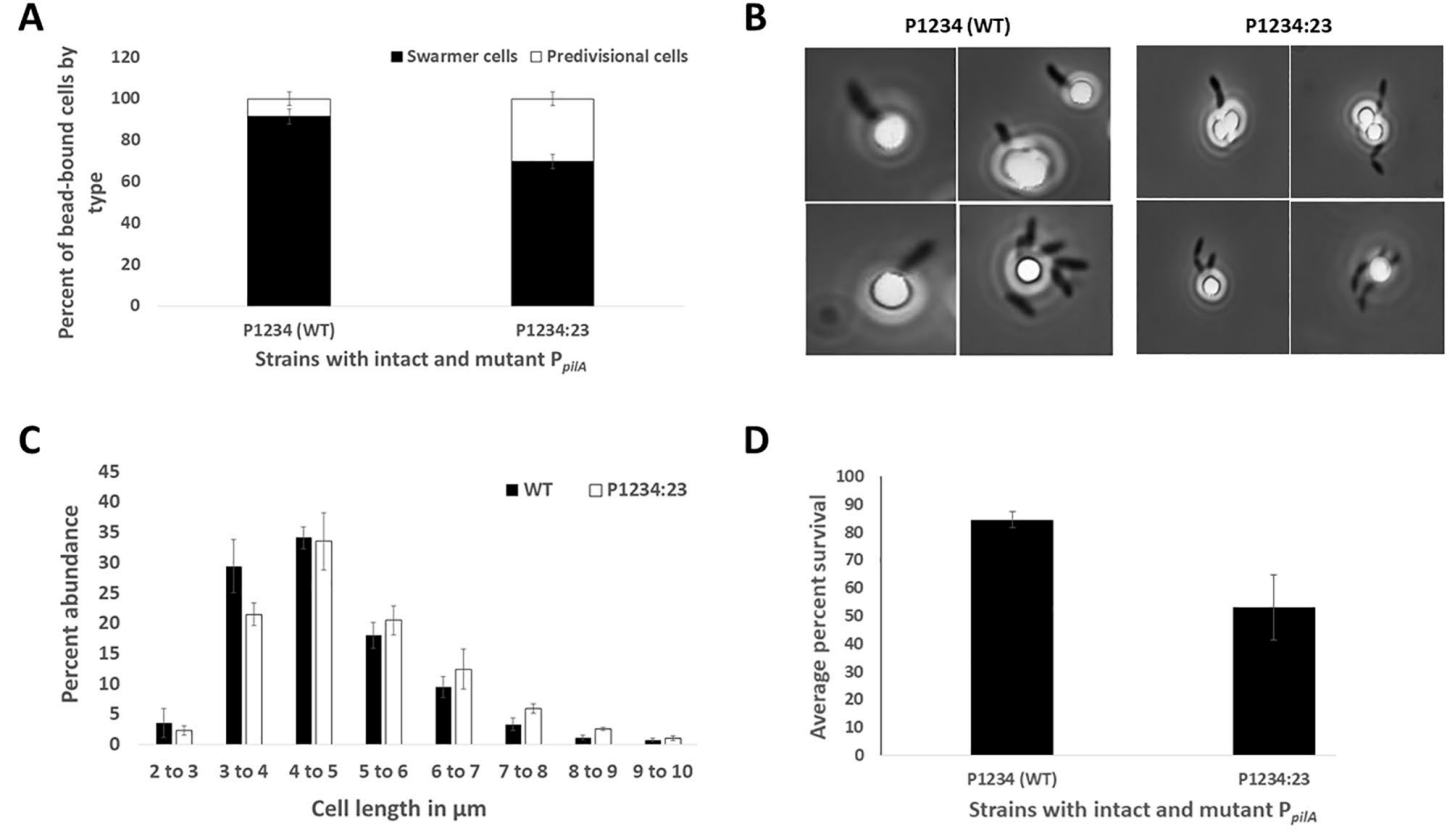
Pilus filament production occurs earlier in P1234:23, increasing sensitivity of *C. crescentus* to phage φCbK. Cells with WT or P1234:23 *pilA* promoters in the *pilA*^*T36C*^ background were treated with biotin-PEG-maleimide to biotinylate the pili. Cells with labeled pili were bound to streptavidin magnetic beads. A: Cells were categorized as swarmer or predivisional based on the absense/presence respectively of a noticeable cell constriction. The P1234:23 strain had a much higher percentage of piliated predivisional cells bound to streptavidin-coated magnetic beads. B: Representative images showing predivisional and swarmer cells bound to magnetic beads. C: Cells were detached from beads and cell length was measured for roughly 7500 cells. Percentage abundance of cells belonging to respective length categories for WT and P1234:23 is shown in a histogram. The percentage abundance of cells of 5 μm or longer was consistently higher for P1234:23, indicating a greater proportion of predivisional cells attached to beads. D. Average percent survival of *C. crescentus* against φCbK infection. WT (P1234) strains showed significantly greater survival compared to P1234:23 with Sites 2 and 3 mutated.

Previous research into pilus gene regulation in *C. crescentus* has shown that pilus assembly proteins are expressed in the mid-predivisional cell stage by GcrA, suggesting the pilus assembly apparatus may be held ready for pilin protein synthesis that occurs later [7, 14, 16, 26]. Indeed, it has been shown that constitutive *pilA* expression can lead to predivisional cells producing pili [14]. In order to determine if the earlier *pilA* transcription and PilA accumulation induced by Sites 2 and 3 mutations (Figs. 5 and 6) actually leads to earlier pilus filament expression, WT (P1234) and mutant (P1234:23) cells, both with the *pilA*^*T36C*^ mutation, were treated with biotin-PEG-maleimide to label pili with biotin and prevent pilus retraction [10]. Cells bearing pili were separated from a mixed population using streptavidin magnetic beads, and the percent of predivisional cells were quantified manually by microscopic observation. No binding of cells to beads was observed with wild-type NA1000 cells lacking the *pilA*^*T36C*^ mutation indicating that the binding to beads only occurs through pili. Cells were categorized as either swarmer or predivisional cells, with predivisional cells having a visible cell constriction. The average percentage of predivisional cells bound to beads was significantly higher for P1234:23 compared to the WT, indicating that Site 2 and 3 mutation does lead to earlier pilus filament expression (Fig. 8A).

To avoid subjective biases in cell stage categorization, in a separate experiment, cells of both strains from three different unsynchronized cultures were bound to streptavidin magnetic beads using a similar procedure as for the assay described above, detached from beads post enrichment, imaged, and cell length was quantified by MicrobeJ [27]. The experiment was done in triplicates and a total of roughly 7500 cells were visualized for each strain. As seen in Fig. 8C, the majority of P1234 cells fell into the 3–5 μm range indicating this was the swarmer cell population. For the P1234:23 (PC0414) strain, there were comparably fewer 3–5 μm sized cells, but consistently more cells of 5 μm and larger (Fig. 8C and Supplementary Figure S15). Accordingly, while the cell lengths of all captured cells were not found to be significantly different between the strains on the overall upon performing a Student’s T-test (*p*-value = 0.5), there were significantly fewer cells in the 3–4 μm range (*p*-value = 0.02) and significantly more cells in the 7–8 μm (*p*-value = 0.01) and 8–9 μm (*p*-value = 0.003) range for P1234:23. There was no statistical difference in cell size distribution as a whole or any individual category when cells from these strains in bulk culture were analyzed without bead enrichment (Supplementary Figure S16). These results suggest that the P1234:23 pili producing cells had a higher proportion of longer cells, consistent with the observation that this strain produced a significantly greater population of predivisional cells attached to the beads. These results together with the previous assays provide further evidence for the hypothesis that mutating Sites 2 and 3 in the *pilA* promoter disrupts the temporal regulation of *pilA* transcription, resulting in a higher population of cells exhibiting earlier pilus filament formation.

### Delayed pilus expression reduces sensitivity of *C. crescentus* to phage φCbK

The data thus far suggests a model where additional upstream CtrA binding sites serve to reduce and, more importantly, delay *pilA* gene expression and pilus biosynthesis to the late predivisional cell stage, essentially ensuring that pilus extension is largely confined to swarmer cells. The selective advantage of this delay is not clear. While pili are important surface attachment organelles for *C. crescentus*, they are also potent targets for bacteriophage, such as φCbK, which principally attaches to the pilus for its infection mechanism. We hypothesize that limiting pilus elaboration on the replicative cell type and presenting pili primarily on the motile swarmer cells may serve to reduce phage infection in the population. In order to test this hypothesis, the survivability of WT *C. crescentus* NA1000 upon exposure to φCbK was compared to the P1234:23 strain where Sites 2 and 3 were mutated. There is no growth rate difference between the two strains, with both having a doubling time of 105 min (Supplementary Figure S17). In the survival assay performed here, cultures were grown to the same optical density, incubated for a short period of time with φCbK, centrifuged to remove residual phage, and plated onto PYE agar plates after appropriate dilution. Total colony forming units compared to those of the same culture without phage exposure allowed calculation of percent survival after phage infection. As seen in Fig. 8D, the WT strain showed 84 +/- 2.75% survival against φCbK, whereas the P1234:23 mutant mutated showed a significantly lower survival rate at 53% +/- 11.63%. This difference in survival rates was found to be significant (*p* < 0.005) using unpaired, two-tailed Student’s T-test. These results suggest that earlier *pilA* expression leads to a larger percentage of the cell population being susceptible to phage infection, which is clearly to the detriment of the bacterium. Importantly, these results also have implications for the lineage survival of this organism in an ecological context (see Discussion).

## Discussion

Transcription of the *pilA* gene is the major decision point for determining when *C. crescentus* produces pili. The pilus assembly apparatus is produced in the mid-predivisional cell stage, induced by the global regulator GcrA, but the separate regulation of the *pilA* gene prevents pili from being extended from the cell surface until cell division. Given the importance of pili not just in surface adhesion but as potent infection targets for bacteriophage, it is not surprising that pili are strictly regulated. This work provides evidence that the *pilA* gene is regulated by a unique mechanism involving the global regulator CtrA. Mutational analysis and biochemical data suggest that CtrA binding to Site 1, which overlaps the − 35 region, drives gene expression, while additional upstream CtrA binding sites serve to repress and delay expression, with Site 2 being the most important inhibitory site. Because the presence or absence of Site 4 did not significantly alter the magnitude or timing of *pilA* expression, the exact role of Site 4 in this regulatory mechanism is unknown. Although CtrA has been mostly known to either activate or repress a given gene in *C. crescentus*, there have been some instances where it has been reported to both activate and repress the same gene. One such example is *ctrA* itself, whose weak P1 promoter is negatively regulated by CtrA and the strong P2 promoter is under positive feedback regulation by CtrA [24]. Activation of the P1 promoter by GcrA leads to a slow increase in CtrA production until it hits a threshold where it represses P1 by binding to a single TTAA half site at its − 10 position and activates the downstream P2 promoter by binding to a near consensus TTAA-N6-TTAA motif overlapping the − 35 position. This leads to a rapid increase in CtrA production. However, this regulation is mediated by two separate promoters and thus is significantly different from *pilA* regulation. Other examples of CtrA having both activating and repressive effects are the *hemE* strong promoter P_s_, the *fliQ*, and the *fliL* promoters; for each promoter, partial depletion of CtrA disrupts repression, but further depletion results in reduction of promoter activity [28]. The cooperative binding aspect of this promoter may hint that cooperative binding is an aspect of the upstream inhibition found in the *pilA* promoter. In the case of the *hemE* P_s_ promoter, CtrA binds to two sites: the consensus TTAA-N7-TTAA motif at the − 35 position and a TTAA-N7-CTAA motif overlapping the − 10 position [25, 28, 29]. High affinity binding of phosphorylated CtrA to the − 35 motif requires cooperative binding with the other motif and strongest repression is observed only when both sites are intact. Transcription from the *hemE* P_s_ is initiated only after the swarmer to stalked transition begins which coincides with the initiation of CtrA dephosphorylation and degradation, with peak activity observed at the stalked cell phase followed by a rapid decline in activity coincident with CtrA accumulation [25]. In the case of *fliQ* and *fliL* promoters, CtrA binds to a single CTAA-N7-TTAA and AAAA-N7-TTAA motif respectively, located at the − 35 position [28]. These genes reach peak expression during the early predivisional cell stage and remain largely repressed for the rest of the cell cycle, although the exact mechanism of repression is unknown [16, 17]. These modes of regulation are all different from the *pilA* promoter in terms of timing of expression and number of CtrA-binding motifs involved. CtrA as a protein is a relatively run-of-the-mill response regulator. These examples show that *C. crescentus* has taken this basic regulatory scheme and customized it in different ways to create more tailored regulatory schemes, with *pilA* regulation the latest example.

Instances of transcription factors binding to multiple sites in the promoter of the same gene and causing both activation and repression have been reported in other bacteria. An example is the Spo0A~P mediated regulation of *sinI* in *Bacillus subtilis* [30]. Nutrient depletion triggers phosphorylation of Spo0A which turns on *sinI* expression, and SinI then binds to and antagonizes the sporulation repressor SinR, thus activating the sporulation pathway. The binding of Spo0A~P to the consensus TTCGACA site centered at the − 46 position (0A box) activates *sinI* transcription, but Spo0A~P also represses *sinI* by binding to 4 additional downstream sites (O1, O2, O3, O4) each of which deviate from the consensus by 2 nucleotides [30]. EMSA experiments showed that at low to intermediate concentrations, Spo0A~P binds to the high affinity 0A box and activates transcription, but at higher concentrations it binds to the low affinity O-boxes and represses the *sinI* promoter [30]. Thus, this mode of regulation uses multiple low-affinity binding sites to induce temporal regulation of the promoter. This is not the case for the *pilA* promoter where the sites with a greater deviation from the consensus (Sites 2 and 3) together have a higher affinity for CtrA and are upstream of the promoter. In *E. coli*, the FNR protein controls the expression of multiple genes in response to oxygen starvation. Generally, FNR binds as a dimer to a 14 bp region with two-half sites 5 bp each at the − 41.5 position and activates transcription by interacting with the C-terminal Domain of the RNAP α-subunit (αCTD) [31]. However, in the *yfiD* promoter known to be activated by FNR, it is known to bind to two such sites with an additional site located at the − 93.5 position, a phenomenon only observed in promoters repressed by FNR [32]. The upstream site was found to downregulate expression even when the downstream activation site was occupied by a FNR dimer [32]. Another study of the *melR* promoter also activated by FNR, reported that the promoter could still be induced by FNR when a second FNR binding site was introduced at many different upstream positions, but a strong repression was observed when the upstream FNR site was between positions − 85 and − 95 [31], indicating that it is the positioning of the upstream binding site that flips the switch from promoter activation to repression. The exact mechanism of repression is unknown.

The mechanism of upstream site inhibition in the *pilA* promoter is not clear. One hypothesis is that the additional upstream sites have higher affinity and titrate CtrA away from the critical Site 1 that drives expression until later stages of the cell cycle when CtrA activity peaks. An alternative hypothesis is that CtrA binding to Site 1 forms an oligomeric complex with CtrA bound to other upstream sites that prevents transcription initiation by preventing CtrA from recruiting RNA polymerase or bending DNA into an unfavorable conformation like the LysR-type transcriptional regulators ArgP in *E. coli* [33] and TsaR in *Comamonas testosteroni* T-2 [34]. It is also possible that the mechanism involves more components than are currently being considered. Mutating Site 3 (P123:3) results in slightly reduced expression, but deleting Site 3 (P12) results in 1.5 times higher activity than the wild type promoter. Similarly, activity for P1 is 1.5 fold higher than WT whereas that for P123:23 is 2.2 fold higher. Other proteins are known to inhibit *pilA* expression, such as SciP or MucR1/2 [35, 36], but the timing of these are tied specifically to the swarmer cell phase and mechanisms of these are controversial. Clearly there is much yet to be discovered about the mechanism of upstream inhibition.

**Fig. 9:**
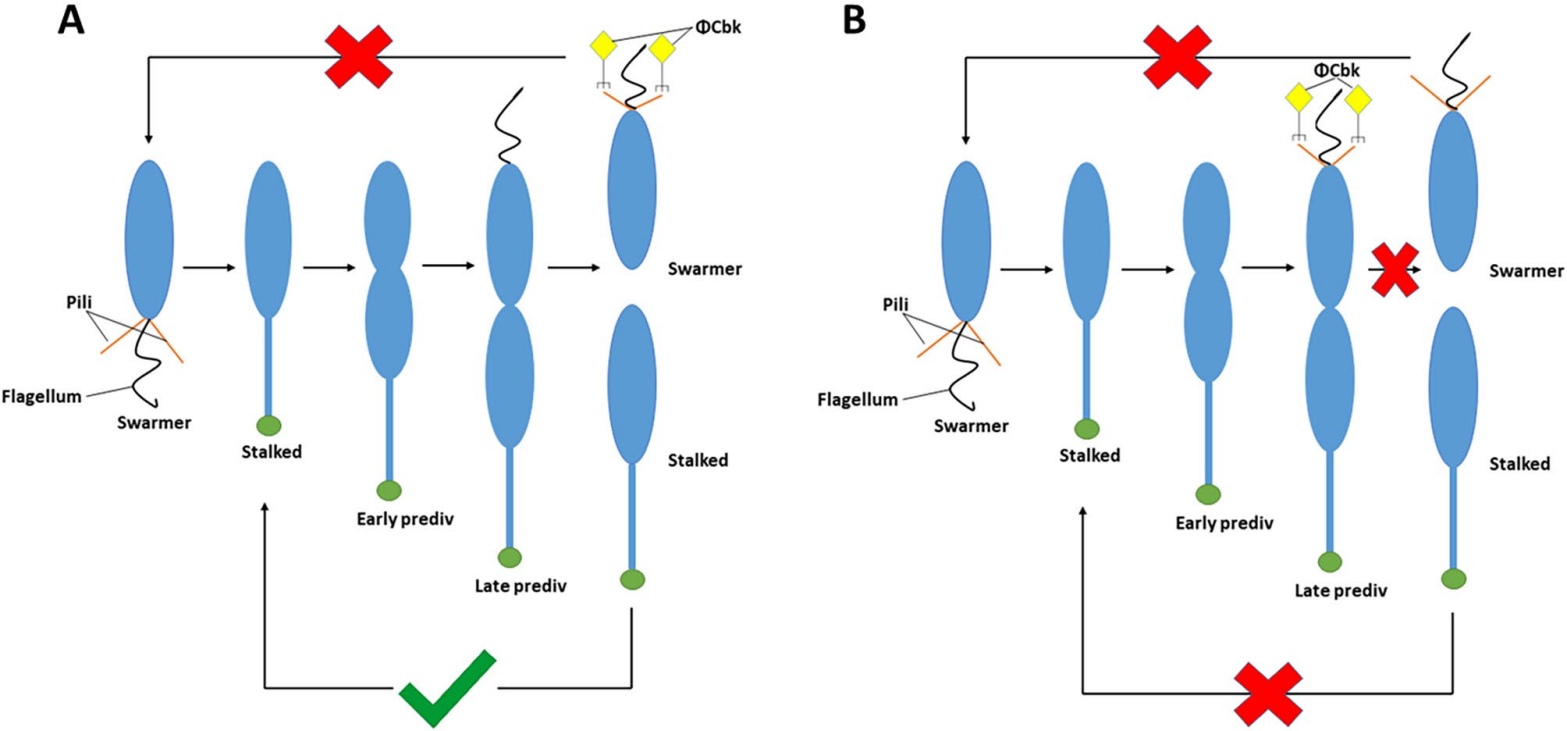
Delayed pilus production prevents termination of *C. crescentus* cell lineages from φCbk phage infection. Schematic representation of the two different scenarios of *C. crescentus* lineage progression after φCbk phage infection based on timing of pilus filament expression. A: In cells with the wild-type *pilA* promoter (P1234), pilus filaments are predominantly expressed after cell division. In this case, φCbk can infect only the swarmer cells and the stalked cell can still continue the lineage by avoiding phage infection. B: In P1234:23 cells with Sites 2 and 3 mutated, a significantly higher proportion of predivisional cells produce pili, which results in φCbk infection before cell division preventing the entire cell lineage from entering another round of cell cycle.

We speculate that, over the course of evolution, acquisition of these upstream inhibitory sites by duplication events and/or point mutations conferred an ecological advantage to *C. crescentus*. Limiting pili to swarmer cells reduces susceptibility to pilitropic bacteriophages such as φCbK. The results from this study showed that survival rate against φCbK decreases significantly for strains where Sites 2 and 3 are mutated (Fig. 8D). In a WT cell population, only swarmer cells are susceptible to phage infection, whereas in the mutant strain a large number of predivisional cells are also susceptible, increasing the total percentage of the population that can be infected. More importantly, these results suggest a long-term ecological advantage to this pilus regulatory scheme, particularly during continued exposure to bacteriophage (Fig. 9). In the natural environment, bacteriophage are likely not transitory, but persistent threats. Limiting pilus biogenesis to the swarmer cell limits phage infection to that cell type, but critically also means that the progenitor mother cell is effectively immune to that phage infection. This means the lineage will be able to persist even under constant phage attack. Newborn swarmer cells may or may not get infected, but the stalked cell and predivisional cells will still be able to grow and divide. The data in Fig. 8 shows that early pilus production leads to predivisional cell susceptibility to the phage. If predivisional cells become infected, that not only kills the cell, but the entire cell lineage. Clearly this mechanism is only relevant to pilitropic Caulophage, but it provides an obvious selectable advantage in the natural environment and can be further explored in the laboratory with more sophisticated mechanisms of tracking cell-phage dynamics.

Comparison of the *pilA* promoter architecture with *Caulobacter segnis*, a close relative of *C. crescentus*, reveals that the region upstream of *pilA* in *C. segnis* has the same four CtrA-binding sites having the same spacing between them as in *C. crescentus*. The conservation of the *pilA* promoter architecture in *C. segnis* might suggest the presence of a similar mode of regulation. While *C. segnis* has also been reported to exhibit a dimorphic lifestyle [37], *pilA* regulation and the timing of pilus formation in *C. segnis* has not been studied. Interestingly, an organism from the sister genus to *Caulobacter*, *Brevundimonas subvibrioides*, also exhibits a dimorphic lifestyle [38, 39], but has a duplicated *pilA* gene, and only one CtrA binding site can be observed in front of only one copy of the gene. However, the more distant Alphaproteobacterium *Agrobacterium tumefaciens* has all six CtrA half-sites in its pilin-encoding *ctp* promoter [40], with one pair of such half-sites placed at a 6 bp spacing and another at a 7 bp spacing. A comparison of the regulation of pilin-encoding genes in these species together with their sensitivities to pilitropic phage might reveal more interesting ecological/evolutionary patterns.

## Materials and methods

### Bacterial strains and growth conditions

*C. crescentus* was grown in solid or liquid Peptone Yeast Extract (PYE) media (2 g/l peptone, 1 g/l yeast extract, 0.3 g/l MgSO_4_. 7H_2_O, 0.0735 g/l CaCl_2_. 2H_2_O, 1.5% agar for plates) at 30 °C. Kanamycin or tetracycline were used when required at final concentrations 20 µg/ml and 2 µg/ml respectively. For synchronization experiments, *C. crescentus* was grown in M2G medium (0.87 g/l Na_2_HPO_4_, 0.54 g/l KH_2_PO_4_, 0.50 g/l NH_4_Cl, 0.2% (w/v) glucose, 0.5 mM MgSO_4_, 0.5mM CaCl_2_, 0.01 mM FeSO_4_). *E. coli* strains were grown in Luria-Bertani (LB) media (10 g/l tryptone, 5 g/l yeast extract, 10 g/l NaCl, 1.5% agar for plates) at 37 °C and kanamycin or tetracycline were used at final concentrations 50 µg/ml and 12.5 µg/ml respectively, when necessary. All strains used in the study have been listed in Table S3.

### Plasmids and strain construction

All plasmids and primer sequences used in the study are listed in Tables S2 and S4 respectively. For creating P_pilA_-*lacZ* fusion strains for β-galactosidase assays, intact or mutant *pilA* promoter fragment sequences were ordered as megaprimers and fused with the promoterless *lacZ* gene in plac290 [41] plasmid using either the T4 DNA ligase (NEB) or Gibson Assembly mastermix (NEB) and the fusion plasmid was inserted into *C. crescentus* NA1000 cells via electroporation [42]. The organization of the different promoter constructs has been illustrated in Table S1.

Allelic replacement was used for creating chromosomal mutations in the *pilA* promoter. Upstream and downstream fragments (approximately 800 bp) from the respective binding sites were PCR-amplified using *C. crescentus* genomic DNA as the template such that either the forward or reverse primer or both introduced the GGCC mutations to the binding sites. These fragments were then cloned into HinDIII and EcoRI-digested pNPTS138 (M.R.K Alley, unpublished) using the Gibson Assembly mastermix to create plasmids pAR0014 (for P1234:1), pAR0015 (for P1234:2), pAR0016 (for P1234:3) and pAR0017 (for P1234:23). The plasmids were then inserted into *C. crescentus* NA1000 *pilA*^*T36C*^ strain via electroporation and the chromosomal mutation was generated via a two-step selection process involving kanamycin resistance and sucrose sensitivity first followed by sucrose resistance and kanamycin sensitivity second. For creating pAR0014, primers P1234:23upF and P1234:1upR were used to amplify the upstream fragment, and primers P1234:1dnF and P1234:23dnR were used to amplify the downstream fragment. To create pAR0015, primers P1234:23upF and P1234:2upR were used to amplify the upstream fragment, and primers P1234:23dnF and P1234:23dnR were used to amplify the downstream fragment. Similarly, to create pAR0016, primers P1234:23upF and P1234:23upR were used to amplify the upstream fragment, and primers P1234:3dnF and P1234:23dnR were used to amplify the downstream fragment. For creating pAR0017, primers P1234:23upF and P1234:23upR were used to amplify the upstream fragment, and primers P1234:23dnF and P1234:23dnR were used to amplify the downstream fragment.

In order to create strain PC0227, phage transduction [43] was performed to transfer the *ctrA* P2 promoter mutation from the CB15 background (strain YB3558) [22] to the NA1000 background. Briefly, 100 µl of φCbK phage stock (10^7^ PFU/ml) was mixed with 200 µl of overnight culture of YB3558 and 3 ml of 0.5% PYE agar, poured onto a PYE agar plate and incubated overnight at 30 °C. PYE (5 ml) was added to the plate the next day and it was incubated at 4 °C overnight. The liquid PYE was harvested off the plate and mixed with 150 µl chloroform in a glass vial. A 1 ml aliquot from that stock was poured onto a petri dish and was irradiated with short-wave UV using a handheld UV device at a 5 cm distance from the plate for 3 min. A 25 µl aliquot of the irradiated lysate was mixed with 500 µl PYE and 475 µl recipient culture (wild-type NA1000), incubated for 3 h in a shaking incubator at 30 °C. 100 µl of this culture was poured onto a PYE-kanamycin plate and incubated for 2 days at 30 °C. Cells were observed under the microscope to check for rosette formation and absence of rosettes confirmed the strain was NA1000.

### β-galactosidase assays

β-galactosidase assays were performed as previously described [22]. Briefly, *C. crescentus* cultures were grown to mid-log phase (OD_600_ = 0.04–0.07). All OD_600_ measurements were done using a Nanodrop spectrophotometer (Thermo Fisher Scientific). A 50 µl sample of culture was mixed with 750 µl Z-buffer, pH 7 (60 mm Na_2_HPO_4_.7H_2_O, 40 mm NaH_2_PO_4_.H2O, 10 mm KCl, 1 mm MgSO_4_.7H_2_O). Chloroform (50 µl) was added and the mixture was incubated at 30 °C for 5 min after vortexing for 5 s. Cold ONPG (200 µl from a 4 mg/ml stock solution) was then added and the time duration for pale yellow coloration to develop was noted. Once the yellow coloration was observed, 400 µl of stop solution (1 M Na_2_CO_3_) was added to stop the reaction. The tubes were centrifuged for 5 min (17,000 x g) at RT and the absorbance of the supernatant at 420 nm (A_420_) was measured. Miller Units were calculated using the formula (A_420_ × 1000)/ [OD_600_ x time in mins x volume of cells (ml)]. The procedure was done 3 times from 3 independent cultures each, and average Miller Units and standard deviation for the 9 replicates were calculated.

To analyze β-galactosidase activity from synchronized cultures, *C. crescentus* cultures were synchronized as previously described [44]. Samples (50 µl) were withdrawn in triplicate from the same culture every 20 min starting from 0 min after synchrony and ending in 180 min. β-galactosidase assays were performed as described above.

### PilA western blotting

For *C. crescentus* strains with chromosomal mutations in the *pilA* promoter, western blotting was performed to measure PilA protein production during the cell cycle. *C. crescentus* cultures were synchronized as previously described [44]. After synchronization, 250 µl of culture was withdrawn every 20 min starting from 0 min after synchrony and ending in 180 min. After centrifugation (2 min, 17,000 x g), the supernatant was removed and the pellet was resuspended in 25 µl 8 M Urea and mixed with 25 µl 4X SDS PAGE sample buffer (0.2 M Tris-HCl, 8% w/v SDS, 6mM bromophenol blue, 4.3 M glycerol, 1% β-mercaptoethanol) after resuspension. The mixture was then boiled at 105 °C for 6 min and 10 µl was loaded into SDS PAGE gels (12% separating gel). After electrophoresis at 180 V for 1 h, transfer of separated bands onto nitrocellulose membrane was performed using a semi-dry electrophoretic transfer cell (Biorad) at 25 V, 1 A for 30 min. Anti-PilA antibody [14, 15] was used at a dilution of 1:5,000, followed by anti-rabbit HRP-conjugated secondary antibody at 1:5,000 dilution and detection of bands was done using Supersignal West Pico chemiluminescence substrate (Pierce), with exposure time of 330 s. Triplicates of synchronized cultures were used for each strain.

Quantification of PilA band-intensity in pixels was performed for all three replicates of each strain using ImageJ software [45], and the average intensity and the standard deviation was calculated for each timepoint for each strain from all three replicates.

### His_6_-CtrA purification and electrophoretic mobility shift assays (EMSA)

For CtrA overexpression and purification, *ctrA* was amplified with primers ctrApET28aF and ctrApET28aR (Table S4) using *C. crescentus* genomic DNA as template and cloned into pET28a using Gibson Assembly to create plasmid pAR001. The plasmid was then inserted into *E. coli* BL21 (DE3) using electroporation to create a CtrA overexpression strain (PC0313) and CtrA purification was done under native conditions using a modified protocol of [46]. A 500 ml culture was grown overnight in an auto-induction medium (6 g/l Na_2_HPO_4_, 3 g/l KH_2_PO_4_, 20 g/l tryptone, 5 g/l yeast extract, 5 g/l NaCl, 0.6% glycerol, 0.05% glucose, 0.2% lactose) at 16 °C [47]. The culture was centrifuged (20,000 x g, 15 min) at 4 °C and the pellet was resuspended in 6 ml lysis buffer [20mM HEPES-KOH (pH 8), 0.5 M NaCl, 10% glycerol, 40 mM Imidazole]. Lysozyme (6 mg) was added and the mixture was incubated for 10 min on ice. Next, cells were lysed by sonication using a Sonic Dismembrator (Fisher Scientific) at 40% amplitude, 7 s pulses with 25 s breaks in between for a total pulse time of 2 min. Following sonication, samples were centrifuged (21,000 x g, 30 min) at 4 °C and the supernatant was mixed with 1 ml of Ni-NTA Agarose slurry (Qiagen) for 1 h on a shaking platform at 4 °C. The mixture was then loaded onto a 1 ml polypropylene column (Qiagen) and flow through was collected for SDS-PAGE. The resin was then washed with 2 ml Wash buffer [20 mM HEPES-KOH (pH 8), 0.5 M NaCl, 10% glycerol, 80 mM Imidazole] and elution was performed with 2 ml Elution buffer [20 mM HEPES-KOH (pH 8), 0.5 M NaCl, 10% glycerol, 100 mM Imidazole]. Four wash samples of 500 µl each were collected separately and mixed with 250 µl glycerol. Four separate elutions of 500 µl each were collected and mixed with 250 µl 100% glycerol prior to storage at -20 °C. Wash and elution samples (10 µl each) were mixed with 10 µl 2X SDS sample buffer, boiled for 10 min at 105 °C and loaded onto 16% SDS-PAGE gels to check for CtrA bands and relative purity of elutions. For MBP-EnvZ purification, the strain YB2351 with the pMal-EnvZ plasmid was obtained [48]. This plasmid was inserted into *E. coli* BL21 (DE3) using electroporation to create strain PC0240. A 500 ml culture of the MBP-EnvZ overexpression strain was grown overnight in auto-induction media (composition described above) at 16 °C. The culture was centrifuged (20,000 x g, 15 min) at 4 °C and the pellet was resuspended in 5 ml column buffer [20 mM Tris-HCl (pH 7.4), 11 g/l NaCl, 1 mM EDTA). Lysozyme (6 mg) was added and the mixture was incubated for 10 min on ice. Next, sonication was performed at 40% amplitude, 7 s pulses with 25 s breaks in between for a total pulse time of 2 min, followed by centrifugation (21,000 x g, 30 min). Amylose resin (2.5 ml, NEB) was loaded onto a 1 ml polypropylene column (Qiagen) and after the flow through was allowed to pass through, the column was washed with 10 ml of column buffer. The lysate was then loaded and after flow through was allowed to pass through, the column was washed again with 10 ml of column buffer. Five 25 µl aliquots of washes were collected for SDS-PAGE analysis. The protein was finally eluted with 3 ml of column buffer supplemented with 10 mM maltose. Six different fractions of 500 µl each were collected and 250 µl of 100% glycerol was added prior to storage at -20 °C. SDS-PAGE was done as described above to check for protein quantity and purity.

For EMSA experiments, CtrA was phosphorylated by making modifications to the protocol described by [49]. Briefly, 7.5 µl CtrA (3.3 µM final concentration) was phosphorylated by incubation with 1 µl ATP (1 mM final concentration) and 1 µl of 7.5 µM MBP-EnvZ (final concentration 0.75 µM) in a buffer consisting of 50 mM Tris HCl (pH 7.8), 50 mM KCl, 20 mM MgCl_2_, 1 mM DTT, at 30 °C for 20 min. Biotin-labeled double-stranded DNA (100 ng) was combined with 2 µl of Binding buffer [50 mM KCl, 5 mM MgCl_2_, 20 mM Tris–HCl (pH 8), 100 µM EDTA, 1 mM DTT, 10% glycerol] and 1 µg Poly dI-dC. Subsequently, specific amounts of phosphorylated CtrA was added to the mixture to get the listed final concentrations (Fig. 7) and the total volume was adjusted to 10 µl. The mixture was then incubated for 25 min at room temperature. Just before loading, 2 µl of loading buffer [100 mM Tris–HCl (pH 7.8), 0.2% bromophenol blue] was added to the mix. A 5% native PAGE gel was prepared with the following composition: 7.5 ml of 1X TBE (pH 8), 5.25 ml water, 2.25 ml 40% polyacrylamide, 6 µl TEMED, 150 µl 10% APS. The gel was pre-run at 150 V for 5 min, and then the samples were loaded. Electrophoresis was carried out at 300 V for 30 min in 1X TBE buffer (pH 8). Subsequently, the gel, blotting paper, and positively charged nylon membrane were incubated in 0.5X TBE buffer (pH 8.3) for 20 min on a shaking platform at 4 °C. Transfer was performed using a semi-dry electrophoretic transfer cell (Biorad) at 25 V, 1 A for 40 min. After transfer, the DNA was crosslinked to the membrane by exposure to UV short wave (254 nm) for 20 min using a handheld UV device. Following this, the membrane was incubated in blocking buffer [1X TBS, pH 7.6 with Tropix I-Block, (Applied Biosystems)] supplemented with 0.2% Tween-20 on a shaking platform for 1 h at room temperature. Subsequently, the membrane was incubated in blocking buffer with 0.2% Tween-20 and 1.5 µl of 1.25 mg/ml HRP-conjugated streptavidin at a dilution of 1:23,000, on a shaking platform for 1 h at room temperature. Five consecutive washes were then conducted, each for 5 min on a shaking platform at room temperature, with 1X TBST (0.2% Tween-20). Finally, the membrane was incubated with 3 ml of Supersignal West Pico PLUS Chemiluminescent Substrate for 10 min before imaging. The EMSAs with CtrA and CtrA∼P were done using the same protocol except that the unphosphorylated CtrA mix didn’t have ATP added to it and that the gel included 10% glycerol.

For calculation of K_d_ values, the protocol described in [50, 51] was modified. The unbound DNA band (bottom band) intensity was quantified in pixels using ImageJ software [45] and was normalized with respect to the bottom band in the ‘DNA only’ lane (Lane 1) to calculate the unbound DNA percentage for each lane. Next, these values were subtracted from 100 to get the respective bound DNA percentage. The software RStudio [52] was used to perform non-linear regression analysis by fitting a model to the protein concentration vs. bound DNA data to calculate the K_d_ values.

### Piliated cell bead binding assay

To selectively enrich for pili producing cells, overnight cultures of strains carrying the *pilA*^*T36C*^ mutation were normalized to OD_600_ of 0.01, grown to OD_600_ of 0.04 in 25 ml PYE and centrifuged (2 min, 5000 x g) at 4 °C. The pellet was resuspended gently in 100 µl PBS (pH 6.8) followed by incubation with 5 µl biotin-PEG-maleimide (BPM) (50 mg/ml in DMSO) for 10 min at room temperature. Cells were centrifuged (2 min, 5000 x g) at RT and after removal of supernatant the pellet was washed with 1 ml PBS (pH 6.8), centrifuged (2 min, 5000 x g) at RT and resuspended in 900 µl PBS (pH 6.8). Meanwhile, 15 µl streptavidin-coated magnetic beads (Dynabeads MyOne Streptavidin T1, ThermoFisher) were washed three times with 1 ml, 500 µl and 200 µl PBS (pH 6.8) respectively with separation on a magnetic stand for 2 min between washes. The final resuspension of beads was done in 100 µl PBS (pH 6.8) and it was mixed with the 900 µl BPM-labelled cells. The cells were allowed to bind to beads while gently shaking for 30 min at room temperature and beads were separated on a magnetic stand for 2 min followed by washing with 1 ml PBS (pH 6.8) twice. The beads with bound cells were used for microscopy for the analysis where cells were quantified and categorized as swarmers or predivisional cells based on the presence of a constriction in the middle.

For the second assay where cells were categorized based on their lengths using ImageJ, the same protocol as described above was used to bind cells to beads. The bead-cell mixture was then incubated on a 55 °C water bath for 5 min followed by vortexing three times for 15 s each to separate the cells from the beads. The beads were separated on a magnetic stand for 2 min and the supernatant with cells was removed and centrifuged (10,000 x g, 5 min) at RT. The pellet was resuspended in 10 µl PBS (pH 6.8) and used for visualization under the microscope using the Metamorph software. Each experiment was performed in triplicate for each strain and brightfield images from each replicate were pooled together for cell length quantification using the MicrobeJ plug-in [27]. Cell length data in pixels was measured which was then converted to µm using an embedded scale in the Metamorph software which was input into MicrobeJ. Values smaller than 2 μm and larger than 12 μm were excluded from the analysis as these came from background noise.

### Phage survival assay

To quantify the survival rate of cells after exposure to φCbK, overnight cultures of WT and P1234:23 strains (*pilA*^*T36C*^ background) were normalized to OD_600_ of 0.01, grown to OD_600_ of 0.04 and diluted 1:1000 in PYE to achieve a final concentration of 10^5^ CFU/ml. A 750 µl aliquot of this diluted culture was mixed with 100 µl of φCbK (10^14^ PFU/ml) in a small petridish (65 mm x 15 mm) and incubated on a shaking platform for 15 min at room temperature to allow for phage adsorption. Post incubation, the culture was centrifuged (2 min, 21,000 x g) at RT and supernatant was removed. Cells were resuspended in 1 ml PYE, diluted 1:100 in PYE and 100 µl was plated on PYE agar. Three separate sets of triplicate plates with and without phage were prepared for each strain, such that there were 3 plates with phage and 3 without in each set for each strain. The CFU/ml was calculated for each set and average CFU/ml with phage and average CFU/ml without phage were calculated. Average CFU/ml with phage was divided by average CFU/ml without phage to get the average percent survival for 1 set. The same was done for all three sets and the overall average percent survival along with standard deviation was calculated such that the final number was an average of averages.

### Electronic supplementary material

Below is the link to the electronic supplementary material.

Supplementary Material 1

## Data Availability

The datasets generated and/or analyzed during the current study are available from the corresponding author on reasonable request.

## References

[1] Fronzes R, Remaut H, Waksman G. Architectures and biogenesis of non-flagellar protein appendages in Gram-negative bacteria. EMBO J. 2008;27(17):2271–80. 18668121 10.1038/emboj.2008.155 PMC2500206

[2] Scott JR, Zähner D. Pili with strong attachments: Gram-positive bacteria do it differently. Mol Microbiol. 2006;62(2):320–30. 16978260 10.1111/j.1365-2958.2006.05279.x

[3] Jarrell KF, Stark M, Nair DB, Chong JPJ. Flagella and pili are both necessary for efficient attachment of Methanococcus maripaludis to surfaces. FEMS Microbiol Lett. 2011;319(1):44–50. 21410509 10.1111/j.1574-6968.2011.02264.x

[4] Proft T, Baker EN. Pili in Gram-negative and Gram-positive bacteria - structure, assembly and their role in disease. Cell Mol Life Sci. 2009;66(4):613–35. 18953686 10.1007/s00018-008-8477-4 PMC11131518

[5] Kaiser D. Bacterial motility: how do pili pull? Curr Biol. 2000;10(21):777–80. 11084348 10.1016/S0960-9822(00)00764-8

[6] Tomich M, Planet PJ, Figurski DH. The tad locus: postcards from the widespread colonization island. Nat Rev Microbiol. 2007;5(5):363–75. 17435791 10.1038/nrmicro1636

[7] Mignolet J, Panis G, Viollier PH. More than a Tad: spatiotemporal control of Caulobacter pili. Curr Opin Microbiol. 2018;42:79–86. 29161615 10.1016/j.mib.2017.10.017

[8] Giltner CL, Nguyen Y, Burrows LL. Type IV Pilin proteins: versatile molecular modules. Microbiol Mol Biol Rev. 2012;76(4):740–72. 23204365 10.1128/MMBR.00035-12 PMC3510520

[9] Kachlany SC, Planet PJ, DeSalle R, Fine DH, Figurski DH, Kaplan JB. Flp-1, the first representative of a new pilin gene subfamily, is required for non-specific adherence of Actinobacillus actinomycetemcomitans. Mol Microbiol. 2001;40(3):542–54. 11359562 10.1046/j.1365-2958.2001.02422.x

[10] Ellison CK, Kan J, Dillard RS, Kysela DT, Ducret A, Berne C, et al. Obstruction of pilus retraction stimulates bacterial surface sensing. Sci (80-). 2017;358(6362):535–8. 10.1126/science.aan5706 PMC5805138 29074778

[11] Matteo Sangermani I, Hug N, Sauter. Thomas Pfohl UJ. Crossm. Am Soc Microbiol. 2019;10(3). 10.1128/mBio.01237-19 PMC6581867 31213565

[12] Goley ED, Iniesta AA, Shapiro L. Cell cycle regulation in Caulobacter: location, location, location. J Cell Sci. 2007;120(20):3501–7. 17928306 10.1242/jcs.005967

[13] Haakonsen DL, Yuan AH, Laub MT. The bacterial cell cycle regulator gcrA is a σ70 cofactor that drives gene expression from a subset of methylated promoters. Genes Dev. 2015;29(21):2272–86. 26545812 10.1101/gad.270660.115 PMC4647560

[14] Skerker JM, Shapiro L. Identification and cell cycle control of a novel pilus system in Caulobacter crescentus. EMBO J. 2000;19(13):3223–34. 10880436 10.1093/emboj/19.13.3223 PMC313932

[15] Viollier PH, Sternheim N, Shapiro L. A dynamically localized histidine kinase controls the asymmetric distribution of polar pili proteins. EMBO J. 2002;21(17):4420–8. 12198144 10.1093/emboj/cdf454 PMC126193

[16] Laub MT, McAdams HH, Feldblyum T, Fraser CM, Shapiro L. Global analysis of the genetic network controlling a bacterial cell cycle. Sci (80-). 2000;290(5499):2144–8. 10.1126/science.290.5499.2144 11118148

[17] Laub MT, Chen SL, Shapiro L, McAdams HH. Genes directly controlled by CtrA, a master regulator of the Caulobacter cell cycle. Proc Natl Acad Sci U S A. 2002;99(7):4632–7. 11930012 10.1073/pnas.062065699 PMC123699

[18] Biondi EG, Reisinger SJ, Skerker JM, Arif M, Perchuk BS, Ryan KR, et al. Regulation of the bacterial cell cycle by an integrated genetic circuit. Nature. 2006;444(7121):899–904. 17136100 10.1038/nature05321

[19] Jacobs C, Domian IJ, Maddock JR, Shapiro L. Cell cycle-dependent polar localization of an essential bacterial histidine kinase that controls DNA replication and cell division. Cell. 1999;97(1):111–20. 10199407 10.1016/S0092-8674(00)80719-9

[20] Angelastro PS, Sliusarenko O, Jacobs-Wagner C. Polar localization of the CckA histidine kinase and cell cycle periodicity of the essential master regulator CtrA in Caulobacter crescentus. J Bacteriol. 2010;192(2):539–52. 19897656 10.1128/JB.00985-09 PMC2805319

[21] Domian IJ, Quon KC, Shapiro L. Cell type-specific phosphorylation and proteolysis of a transcriptional regulator controls the G1-to-S transition in a bacterial cell cycle. Cell. 1997;90(3):415–24. 9267022 10.1016/S0092-8674(00)80502-4

[22] Curtis PD, Klein D, Brun YV. Effect of a ctrA promoter mutation, causing a reduction in CtrA abundance, on the cell cycle and development of Caulobacter crescentus. BMC Microbiol [Internet]. 2013;13(1):1. Available from: BMC Microbiology. 10.1186/1471-2180-13-166 PMC3751295 23865946

[23] Wortinger M, Sackett MJ, Brun YV. CtrA mediates a DNA replication checkpoint that prevents cell division in Caulobacter crescentus. EMBO J. 2000;19(17):4503–12. 10970844 10.1093/emboj/19.17.4503 PMC302065

[24] Domian IJ, Reisenauer A, Shapiro L. Feedback control of a master bacterial cell-cycle regulator. Proc Natl Acad Sci U S A. 1999;96(12):6648–53. 10359766 10.1073/pnas.96.12.6648 PMC21969

[25] Marczynski GT, Lentine K, Shapiro L. A developmentally regulated chromosomal origin of replication uses essential transcription elements. Genes Dev. 1995;9(12):1543–57. 7601356 10.1101/gad.9.12.1543

[26] Murray SM, Panis G, Fumeaux C, Viollier PH, Howard M. Computational and genetic reduction of a cell cycle to its Simplest, Primordial Components. PLoS Biol. 2013;11(12). 10.1371/journal.pbio.1001749 PMC3885167 24415923

[27] Ducret A, Quardokus EM, Brun YV. MicrobeJ, a tool for high throughput bacterial cell detection and quantitative analysis. Nat Microbiol [Internet]. 2016;1(7):1–7. 10.1038/nmicrobiol.2016.77. 10.1038/nmicrobiol.2016.77 PMC5010025 27572972

[28] Quon KC, Marczynski GT, Shapiro L. Cell cycle control by an essential bacterial two-component signal transduction protein. Cell. 1996;84(1):83–93. 8548829 10.1016/S0092-8674(00)80995-2

[29] Siam R, Marczynski GT. Cell cycle regulator phosphorylation stimulates two distinct modes of binding at a chromosome replication origin. EMBO J. 2000;19(5):1138–47. 10698954 10.1093/emboj/19.5.1138 PMC305652

[30] Chai Y, Norman T, Kolter R, Losick R. Evidence that metabolism and chromosome copy number control mutually exclusive cell fates in Bacillus subtilis. EMBO J [Internet]. 2011;30(7):1402–13. 10.1038/emboj.2011.36. 10.1038/emboj.2011.36 PMC3094124 21326214

[31] Barnard AML, Green J, Busby SJW. Transcription regulation by tandem-bound FNR at Escherichia coli promoters. J Bacteriol. 2003;185(20):5993–6004. 14526010 10.1128/JB.185.20.5993-6004.2003 PMC225037

[32] Green J, Baldwin ML, Richardson J. Downregulation of Escherichia coli yfiD expression by FNR occupying a site at -93.5 involves the AR1-containing face of FNR. Mol Microbiol. 1998;29(4):1113–23. 9767578 10.1046/j.1365-2958.1998.01002.x

[33] Le Nguyen P, Velázquez Ruiz C, Vandermeeren S, Abwoyo P, Bervoets I, Charlier D. Differential protein-DNA contacts for activation and repression by ArgP, a LysR-type (LTTR) transcriptional regulator in Escherichia coli. Microbiol Res [Internet]. 2018;206(September 2017):141–58. 10.1016/j.micres.2017.10.009. 10.1016/j.micres.2017.10.009 29146251

[34] Monferrer D, Tralau T, Kertesz MA, Dix I, Solà M, Usón I. Structural studies on the full-length LysR-type regulator TsaR from Comamonas testosteroni T-2 reveal a novel open conformation of the tetrameric LTTR fold. Mol Microbiol. 2010;75(5):1199–214. 20059681 10.1111/j.1365-2958.2010.07043.x

[35] Gora KG, Tsokos CG, Chen YE, Srinivasan BS, Perchuk BS, Laub MT. A cell-type-specific protein-protein interaction modulates transcriptional activity of a master regulator in caulobacter crescentus. Mol Cell [Internet]. 2010;39(3):455–67. 10.1016/j.molcel.2010.06.024. 10.1016/j.molcel.2010.06.024 PMC3073018 20598601

[36] Fumeaux C, Radhakrishnan SK, Ardissone S, Théraulaz L, Frandi A, Martins D et al. Cell cycle transition from S-phase to G1 in Caulobacter is mediated by ancestral virulence regulators. Nat Commun. 2014;5(May). 10.1038/ncomms5081 PMC4083442 24939058

[37] Patel S, Fletcher B, Scott DC, Ely B. Genome sequence and phenotypic characterization of Caulobacter segnis. Curr Microbiol [Internet]. 2015;70(3):355–63. http://link.springer.com/10.1007/s00284-014-0726-1. 10.1007/s00284-014-0726-1 25398322

[38] Scott D, Ely B. Conservation of the Essential Genome Among Caulobacter and Brevundimonas Species. Curr Microbiol [Internet]. 2016;72(5):503–10. http://link.springer.com/10.1007/s00284-015-0964-x. 10.1007/s00284-015-0964-x PMC4829470 26750121

[39] Curtis PD, Brun YV. Identification of essential alphaproteobacterial genes reveals operational variability in conserved developmental and cell cycle systems. Mol Microbiol. 2014;93(4):713–35. 24975755 10.1111/mmi.12686 PMC4132054

[40] Wang Y, Haitjema CH, Fuqua C. The Ctp type IVb pilus locus of Agrobacterium tumefaciens directs formation of the common pili and contributes to reversible surface attachment. J Bacteriol [Internet]. 2014;196(16):2979–88. 10.1128/JB.01670-14. 10.1128/JB.01670-14 PMC4135632 24914181

[41] Gober JW, Shapiro L. A developmentally regulated Caulobacter flagellar promoter is activated by 3′ enhancer and IHF binding elements. Mol Biol Cell. 1992;3(8):913–26. 1392079 10.1091/mbc.3.8.913 PMC275648

[42] Ely B. Genetics of Caulobacter crescentus. ScienceDirect. 2004;204:372–84. 10.1016/0076-6879(91)04019-k 1658564

[43] Ely B, Johnson RC. Generalized transduction in Caulobacter crescentus. Genetics. 1977;87(3):391–9. 17248770 10.1093/genetics/87.3.391 PMC1213749

[44] Schrader JM, Shapiro L. Synchronization of Caulobacter crescentus for investigation of the bacterial cell cycle. J Vis Exp. 2015;2015(98):1–6. 10.3791/52633 PMC4541484 25938623

[45] Schindelin J, Arganda-Carrera I, Frise E, Verena K, Mark L, Tobias P et al. Fiji - an open platform for biological image analysis. Nat Methods. 2009;9(7). 10.1038/nmeth.2019 PMC3855844 22743772

[46] Skerker JM, Prasol MS, Perchuk BS, Biondi EG, Laub MT. Two-component signal transduction pathways regulating growth and cell cycle progression in a bacterium: a system-level analysis. PLoS Biol. 2005;3(10). 10.1371/journal.pbio.0030334 PMC1233412 16176121

[47] Studier FW. Protein production by auto-induction in high density shaking cultures. Protein Expr Purif. 2005;41(1):207–34. 15915565 10.1016/j.pep.2005.01.016

[48] Kelly AJ, Sackett MJ, Din N, Quardokus E, Brun YV. Cell cycle-dependent transcriptional and proteolytic regulation of FtsZ in Caulobacter. Genes Dev. 1998;12(6):880–93. 9512521 10.1101/gad.12.6.880 PMC316630

[49] Reisenauer A, Quon K, Shapiro L. The CtrA response regulator mediates temporal control of gene expression during the Caulobacter cell cycle. J Bacteriol. 1999;181(8):2430–9. 10198005 10.1128/JB.181.8.2430-2439.1999 PMC93667

[50] Minguk Seo, Li Lei ME. Label-free Electrophoretic mobility Shift Assay (EMSA) for measuring dissociation constants of Protein-RNA complexes. Physiol Behav. 2016;176(1):139–48. 10.1002/cpnc.70 PMC6391183 30461222

[51] Heffler MA, Walters RD, Kugel JF. Using electrophoretic mobility shift assays to measure equilibrium dissociation constants: GAL4-p53 binding DNA as a model system. Biochem Mol Biol Educ [Internet]. 2012;40(6):383–7. https://iubmb.onlinelibrary.wiley.com/doi/10.1002/bmb.20649. 10.1002/bmb.20649 23166026

[52] R Core Team. (2023). _R: A Language and Environment for Statistical Computing_. R Foundation for Statistical Computing, Vienna, Austria. https://www.R-project.org/.

