# Supplementary material for: Upstream CtrA-binding sites both induce and repress pilin gene expression in Caulobacter crescentus
Source: BMC Genomics. 2024 Jul 19;25:703. doi: 10.1186/s12864-024-10533-6 (PMC11264516; doi:10.1186/s12864-024-10533-6)
Supplement: Supplementary file 1 — Supplementary Material 1 [file 12864_2024_10533_MOESM1_ESM.docx]

| **P*_pilA_*-*lacZ* construct name** | **Construct design** | **Description** |
| --- | --- | --- |
| P1234 | 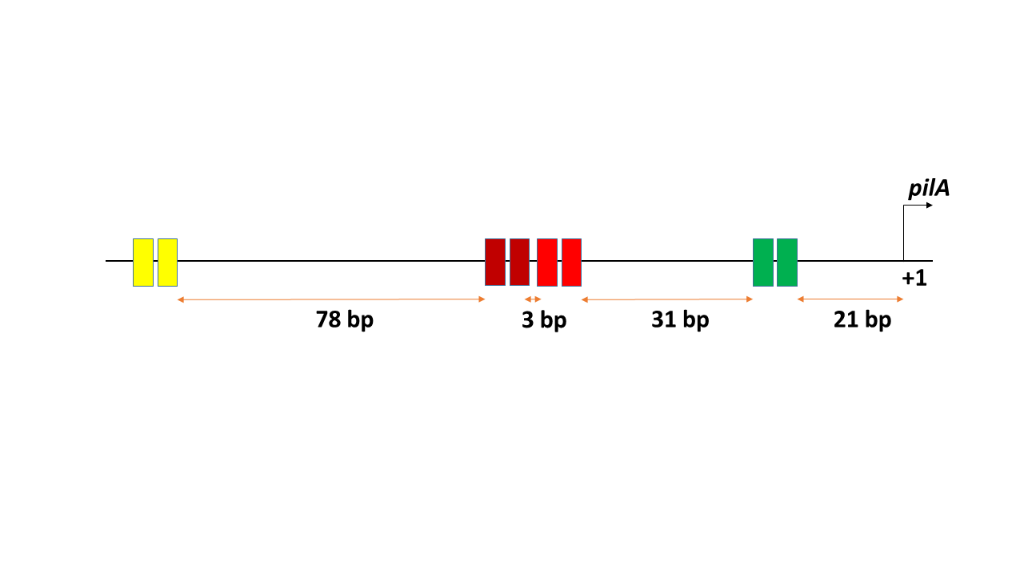 | Wild Type pilA promoter organization. Site 1 (green) is at -35, Site 2 (red) is 31 bp upstream of Site1, Site 3 (maroon) is 3 bp upstream of Site 2, Site 4 (yellow) is 78 bp upstream of Site 3. |
| P123 | 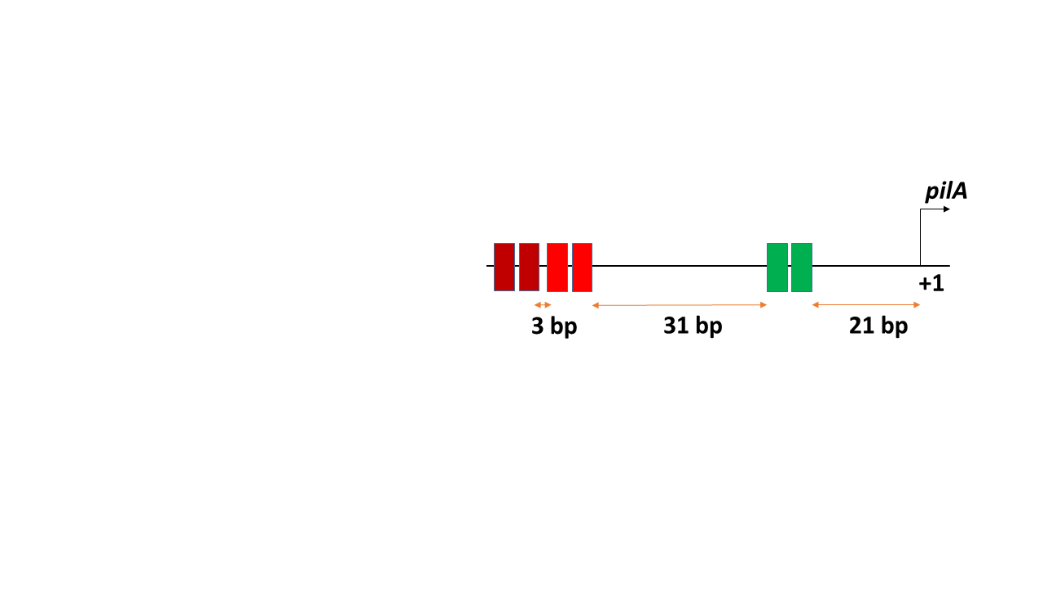 | Site 4 deleted. |
| P12 | 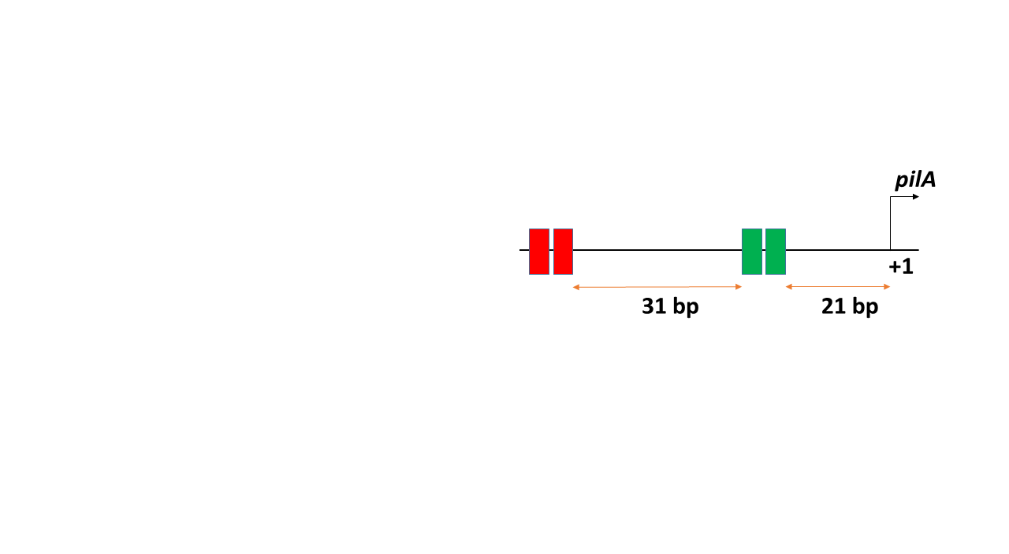 | Sites 2 and 3 deleted. |
| P1 | 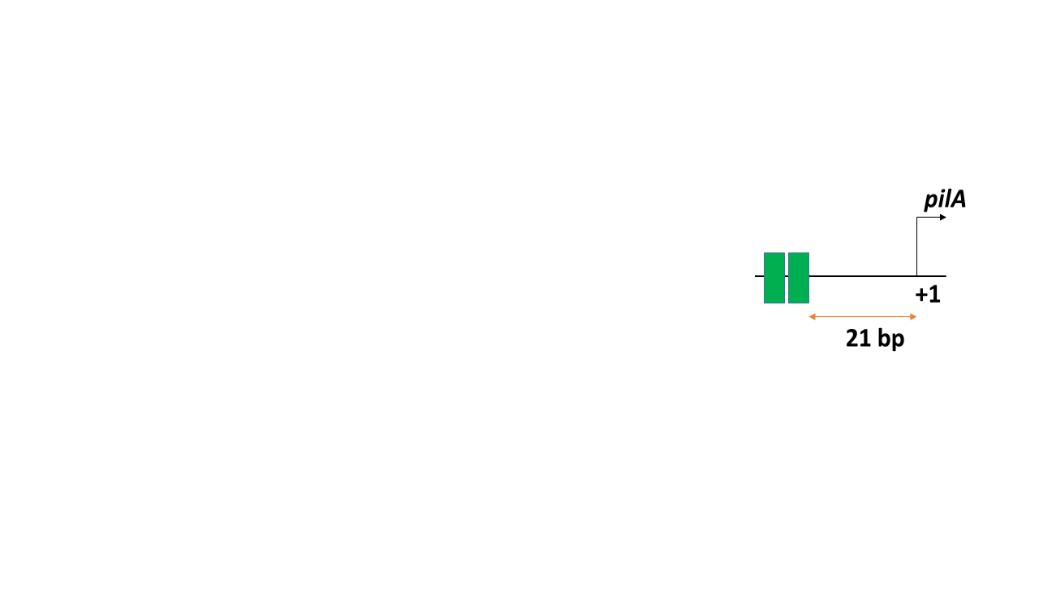 | Sites 2, 3 and 4 deleted. |
| P123:1 | 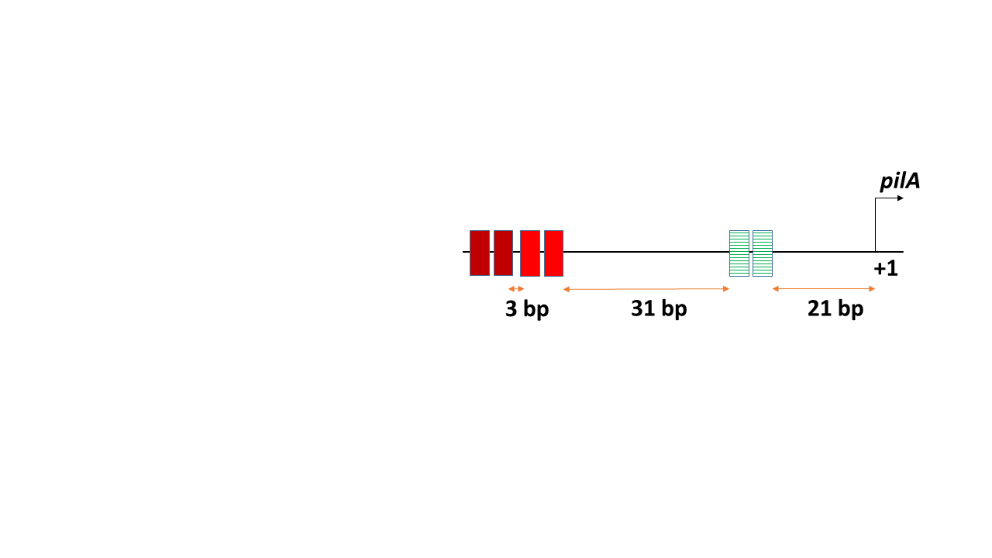 | Site 4 deleted. Site 1 mutated to GGCC. |
| P123:2 | 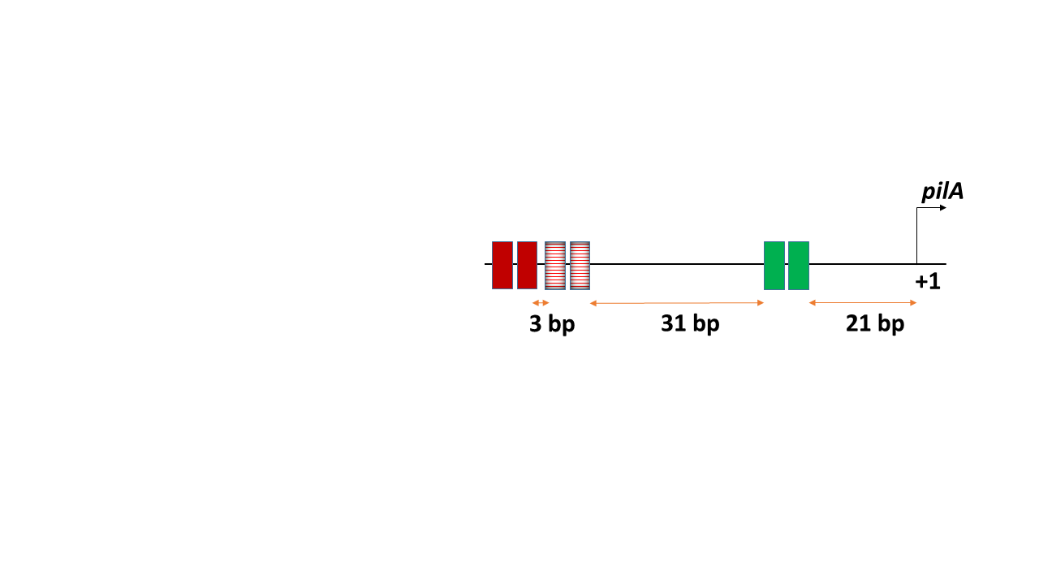 | Site 4 deleted. Site 2 mutated to GGCC. |
| P123:3 | 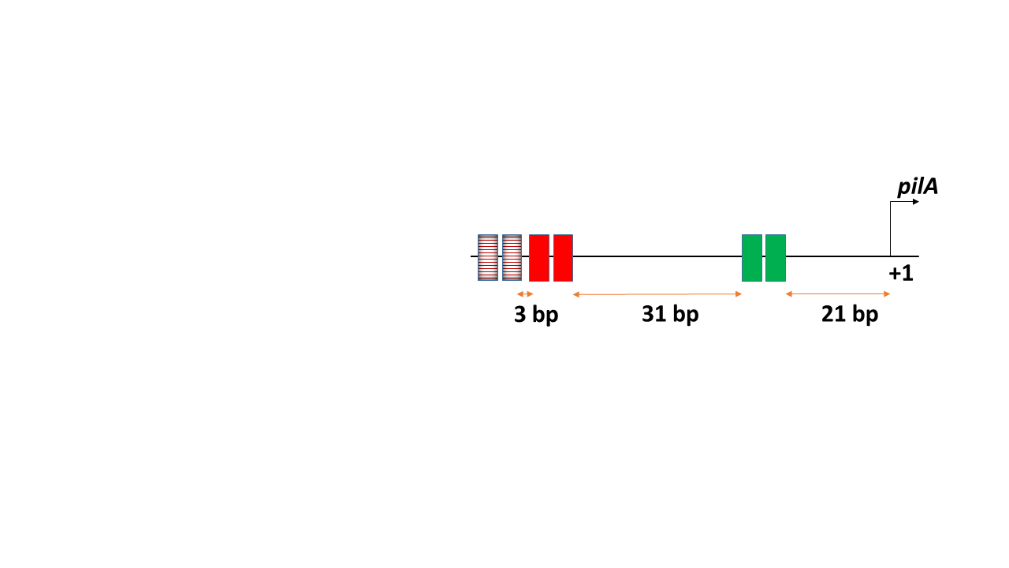 | Site 4 deleted. Site 3 mutated to GGCC. |
| P123:23 | 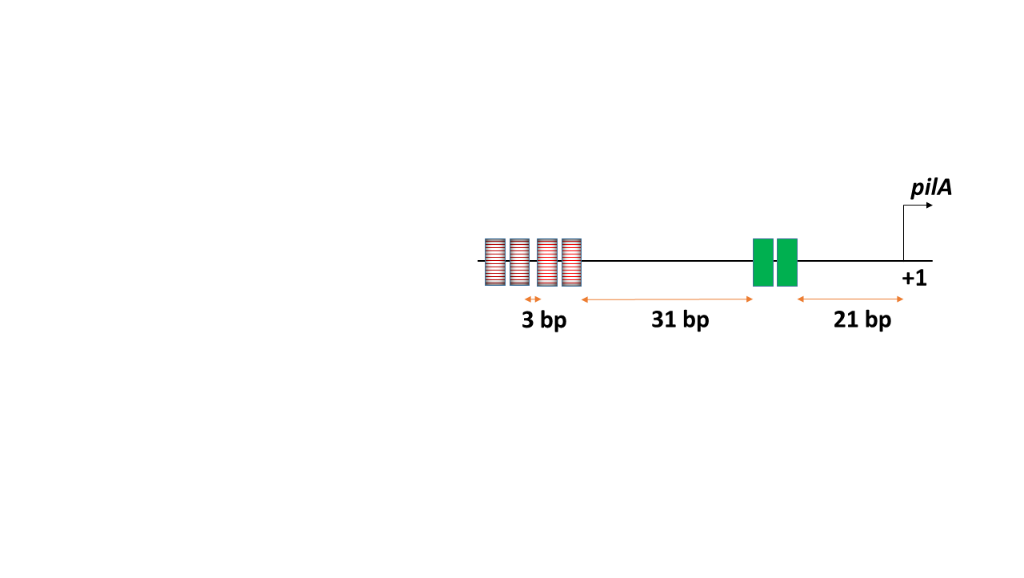 | Site 4 deleted. Sites 2 and 3 mutated to GGCC. |
| P1(2X)N | 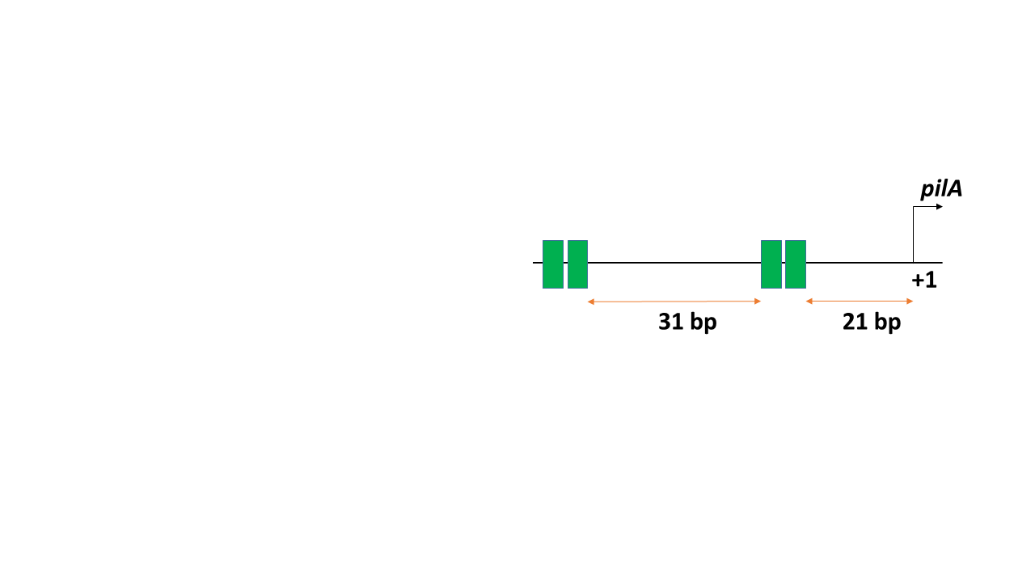 | Site 2, 3, 4 deleted. An extra copy of Site 1 placed at native Site 2 position. |
| P1(3X)N | 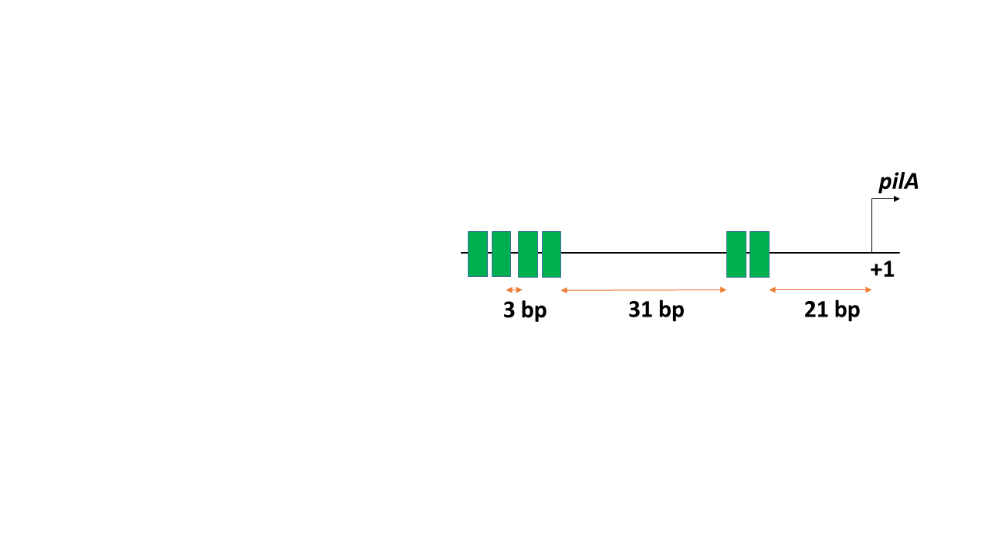 | Sites 2, 3, 4 deleted. Two extra copies of Site 1 used to replace Sites 2 and 3 at their native positions. |
| P1(2X) | 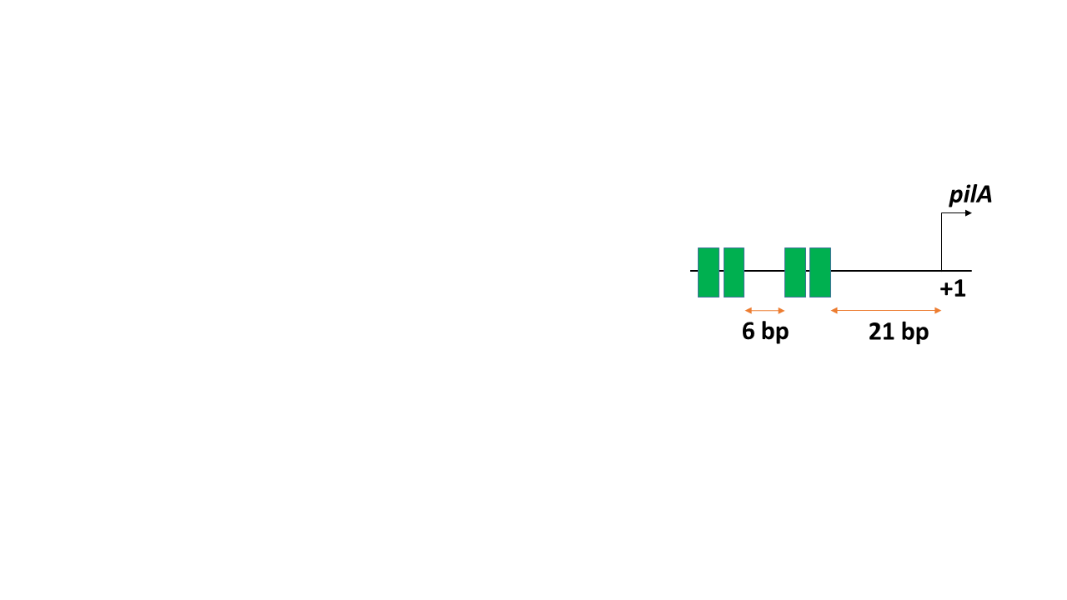 | Sites 2, 3, 4 deleted. An extra copy of Site 1 placed 6bp upstream of native Site 1 position. |
| P1(3X) | 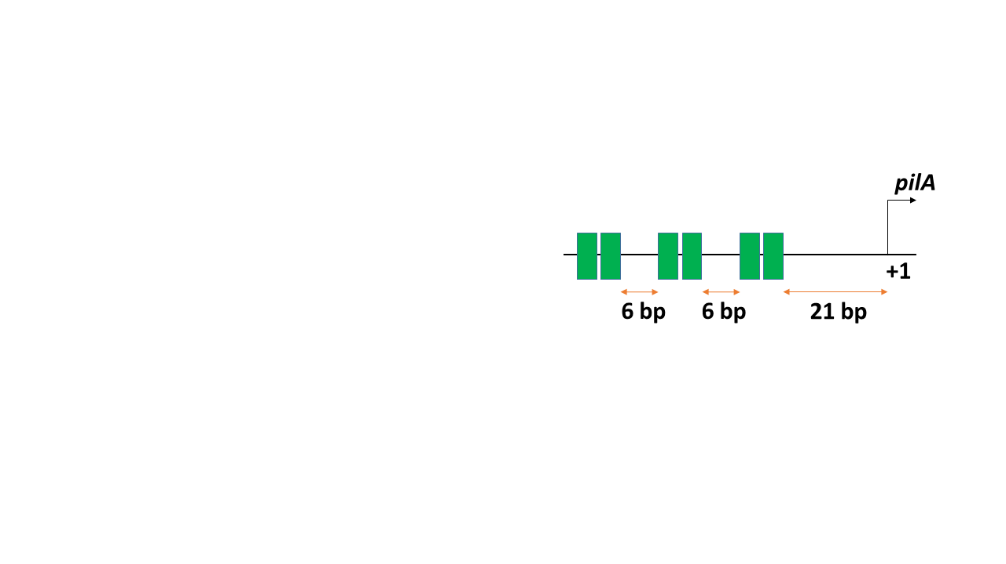 | Similar to construct P1(2X), except that a third copy of Site 1 placed 6bp upstream of the second copy. |
| P1(3X)31 | 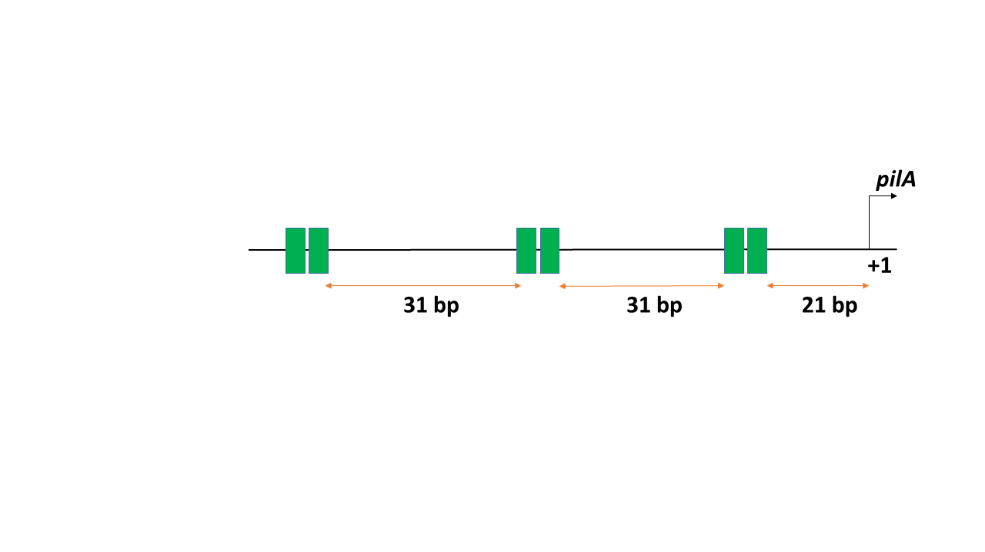 | Similar to construct P1(2X)N, except that a third copy of Site 1 added 31bp upstream of the second copy. |

**Table S1: Schematic representation of the P*_pilA_* architecture in the intact and mutated P*_pilA_*-*lacZ* constructs used for β-galactosidase assay in *C. crescentus* NA1000. Site 1 nucleotide sequence is indicated in green, Site 2 in red, Site 3 in maroon and Site 4 in yellow.**

| **Plasmid** | **Description** | **Source or reference** |
| --- | --- | --- |
| pAR0001 | pET28a-ctrA, Kan^R^ | This study |
| pAR0002 | plac290-P12 (P*_pilA_*), tet^R^ | This study |
| pAR0003 | plac290-P123:1 (P*_pilA_*), tet^R^ | This study |
| pAR0004 | plac290-P123:2 (P*_pilA_*), tet^R^ | This study |
| pAR0005 | plac290-P123:23 (P*_pilA_*), tet^R^ | This study |
| pAR0006 | plac290-P1(2X)N (P*_pilA_*), tet^R^ | This study |
| pAR0007 | plac290-P1(3X)N (P*_pilA_*), tet^R^ | This study |
| pAR0008 | plac290-P1(2X) (P*_pilA_*), tet^R^ | This study |
| pAR0009 | plac290-P1(3X) (P*_pilA_*), tet^R^ | This study |
| pAR0010 | plac290-P1(3X)31 (P*_pilA_*), tet^R^ | This study |
| pNPTS138 | Kan^R^ | M.R.K. Alley, unpublished |
| pAR0014 | pNPTS138-P1234:1, Kan^R^ | This study |
| pAR0015 | pNPTS138-P1234:2, Kan^R^ | This study |
| pAR0016 | pNPTS138-P1234:3, Kan^R^ | This study |
| pAR0017 | pNPTS138-P1234:1, Kan^R^ | This study |
| pSC1 | plac290-P123 (P*_pilA_*), tet^R^ | This study |
| pSC2 | plac290-P1 (P*_pilA_*), tet^R^ | This study |
| pSC3 | plac290-P123:3 (P*_pilA_*), tet^R^ | This study |
| pSC4 | plac290-P1234 (P*_pilA_*), tet^R^ | This study |
| pKJH5 | pMBP-EnvZ, Amp^R^ | (1) |

**Table S2: List of plasmids used in this study.**

| **Strain** | **Description** | **Source or reference** |
| --- | --- | --- |
| *C. crescentus NA1000* | Synchronizable derivative of CB15 | (2) |
| *E. coli* | DH5α-general cloning strain |  |
| YB8288 | *Cc* NA1000 *pilA^T36C^* | (3) |
| PC0313 | pET28a-*ctrA,* Kan^R^ | This study |
| PC0240 | BL21λDE3; pKJH5, Amp^R^ |  |
| YB3558 | *Cc* CB15 P_ctrA_::Mn Kan^R^ | (4) |
| PC0227 | *Cc* NA1000 P_ctrA_::Mn Kan^R^ | This study |
| PC0311 | *Cc* NA1000 / plac290-P*_pilA_* (P12) tet^R^ | This study |
| PC0469 | *Cc* NA1000 P_ctrA_::Mn/ plac290- P*_pilA_* (P12) tet^R^  Kan^R^ | This study |
| PC0223 | *Cc* NA1000/ plac290-P*_pilA_* (P123:1) tet^R^ | This study |
| PC0234 | *Cc* NA1000 P_ctrA_::Mn/ plac290-P*_pilA_* (P123:1) tet^R^  Kan^R^ |  |
| PC0164 | *Cc* NA1000 / plac290- P*_pilA_* (P123:2) tet^R^ | This study |
| PC0232 | *Cc* NA1000 P_ctrA_::Mn/plac290-P*_pilA_* (P123:2) tet^R^  Kan^R^ | This study |
| PC0161 | *Cc* NA1000 / plac290- P*_pilA_* (P123:23) tet^R^ | This study |
| PC0231 | *Cc* NA1000 P_ctrA_::Mn / plac290-P*_pilA_* (P123:23) tet^R^  Kan^R^ | This study |
| PC0285 | *Cc* NA1000 / plac290-P*_pilA_* [P1(2X)N] tet^R^ | This study |
| PC0470 | *Cc* NA1000 P_ctrA_::Mn / plac290-P*_pilA_* [P1(2X)N] tet^R^ Kan^R^ | This study |
| PC0287 | *Cc* NA1000 / plac290-P*_pilA_* [P1(3X)N] tet^R^ | This study |
| PC0471 | *Cc* NA1000 P_ctrA_::Mn / plac290-P*_pilA_* [P1(3X)N] tet^R^ Kan^R^ | This study |
| PC0221 | *Cc* NA1000 / plac290-P*_pilA_* [P1(2X)] tet^R^ | This study |
| PC0235 | *Cc* NA1000 P_ctrA_::Mn / plac290-P*_pilA_* [P1(2X)] tet^R^ Kan^R^ | This study |
| PC0222 | *Cc* NA1000 / plac290-P*_pilA_* [P1(3X)] tet^R^ | This study |
| PC0236 | *Cc* NA1000 P_ctrA_::Mn / plac290-P*_pilA_* [P1(3X)] tet^R^ Kan^R^ | This study |
| PC0308 | *Cc* NA1000 / plac290-P*_pilA_* [P1(3X)31] tet^R^ | This study |
| PC0472 | *Cc* NA1000 P_ctrA_::Mn / plac290-P*_pilA_* [P1(3X)31] tet^R^ Kan^R^ | This study |
| PC0147 | *Cc* NA1000 / plac290-P*_pilA_* (P1234) tet^R^ | This study |
| PC0228 | *Cc* NA1000 P_ctrA_::Mn / plac290-P*_pilA_* (P1234) tet^R^ Kan^R^ | This study |
| PC0146 | *Cc* NA1000 / plac290-P*_pilA_* (P123) tet^R^ | This study |
| PC0229 | *Cc* NA1000 P_ctrA_::Mn / plac290-P*_pilA_* (P123) tet^R^ Kan^R^ | This study |
| PC0145 | *Cc* NA1000 / plac290-P*_pilA_* (P1) tet^R^ | This study |
| PC0230 | *Cc* NA1000 P_ctrA_::Mn/ plac290-P*_pilA_* (P1) tet^R^ Kan^R^ | This study |
| PC0148 | *Cc* NA1000 / plac290-P*_pilA_* (P123:3) tet^R^ | This study |
| PC0233 | *Cc* NA1000 P_ctrA_::Mn / plac290-P*_pilA_* (P123:3) tet^R^ Kan^R^ | This study |
| PC0467 | *Cc* NA1000 P*_pilA_* (P1234:1), *pilA^T36C^* | This study |
| PC0468 | *Cc* NA1000 P*_pilA_* (P1234:2), *pilA^T36C^* | This study |
| PC0465 | *Cc* NA1000 P*_pilA_* (P1234:3), *pilA^T36C^* | This study |
| PC0414 | *Cc* NA1000 P*_pilA_* (P1234:23), *pilA^T36C^* | This study |
| PC0385 | *Cc* NA1000 P*_pilA_* (P1234:23) | This study |
| PC0485 | *Cc* NA1000 / plac290-P*_xyl_* tet^R^ | This study |
| PC0486 | *Cc* NA1000 P_ctrA_::Mn / plac290-P*_xyl_* tet^R^  Kan^R^ | This study |

**Table S3: List of strains used in this study.**

| **Primers** | **Nucleotide sequence** | **Purpose** |
| --- | --- | --- |
| P1F | aattcATTCGCTGTTTACTGGCCATTAAGTGCAGTCGGCGAAATTGATCa | F primer ordered as megaprimer to create P1 construct for β-gal assay |
| P1R | agcttGATCAATTTCGCCGACTGCACTTAATGGCCAGTAAACAGCGAATg | R primer ordered as megaprimer to create P1 construct for β-gal assay |
| P123F | aattcGGAGTGCATGGTTAAGAACAAATAACGGTAAATACAAATAAACCAAAAGAAAATCTCTGAAAACTATTCGCTGTTTACTGGCCATTAAGTGCAGTCGGCGAAATTGATCa | F primer ordered as megaprimer to create P123 construct for β-gal assay |
| P123R | agcttGATCAATTTCGCCGACTGCACTTAATGGCCAGTAAACAGCGAATAGTTTTCAGAGATTTTCTTTTGGTTTATTTGTATTTACCGTTATTTGTTCTTAACCATGCACTCCg | R primer ordered as megaprimer to create P123 construct for β-gal assay |
| P123:1F | GAACCCGCTGAATGGGAATTCGGAGTGCATGGTTAAGAACAAATAACGGTAAATACAAATAAACCAAAAGAAAATCTCTGAAAACTATTCGCTGCCGGCTGGCCACCGGGTGCAGTCGGCGAAATTGATCAAGCTTTCACACAGGAAACA | F primer ordered as megaprimer to create P123:1 construct for β-gal assay |
| P123:1R | TGTTTCCTGTGTGAAAGCTTGATCAATTTCGCCGACTGCACCCGGTGGCCAGCCGGCAGCGAATAGTTTTCAGAGATTTTCTTTTGGTTTATTTGTATTTACCGTTATTTGTTCTTAACCATGCACTCCGAATTCCCATTCAGCGGGTTC | R primer ordered as megaprimer to create P123:1 construct for β-gal assay |
| P123:2F | GAACCCGCTGAATGGGAATTCGGAGTGCATGGTTAAGAACAAATAACGGGGCCTACAAAGGCCCCAAAAGAAAATCTCTGAAAACTATTCGCTGTTTACTGGCCATTAAGTGCAGTCGGCGAAATTGATCAAGCTTTCACACAGGAAACA | F primer ordered as megaprimer to create P123:2 construct for β-gal assay |
| P123:2R | TGTTTCCTGTGTGAAAGCTTGATCAATTTCGCCGACTGCACTTAATGGCCAGTAAACAGCGAATAGTTTTCAGAGATTTTCTTTTGGGGCCTTTGTAGGCCCCGTTATTTGTTCTTAACCATGCACTCCGAATTCCCATTCAGCGGGTTC | R primer ordered as megaprimer to create P123:2 construct for β-gal assay |
| P123:3F | aattcGGAGTGCATGGGGCCGAACAAGGCCCGGTAAATACAAATAAACCAAAAGAAAATCTCTGAAAACTATTCGCTGTTTACTGGCCATTAAGTGCAGTCGGCGAAATTGATCa | F primer ordered as megaprimer to create P123:3 construct for β-gal assay |
| P123:3R | agcttGATCAATTTCGCCGACTGCACTTAATGGCCAGTAAACAGCGAATAGTTTTCAGAGATTTTCTTTTGGTTTATTTGTATTTACCGGGCCTTGTTCGGCCCCATGCACTCCg | R primer ordered as megaprimer to create P123:3 construct for β-gal assay |
| P123:23F | GAACCCGCTGAATGGGAATTCGGAGTGCATGGGGCCGAACAAGGCCCGGGGCCTACAAAGGCCCCAAAAGAAAATCTCTGAAAACTATTCGCTGTTTACTGGCCATTAAGTGCAGTCGGCGAAATTGATCAAGCTTTCACACAGGAAACA | F primer ordered as megaprimer to create P123:23 construct for β-gal assay |
| P123:23R | TGTTTCCTGTGTGAAAGCTTGATCAATTTCGCCGACTGCACTTAATGGCCAGTAAACAGCGAATAGTTTTCAGAGATTTTCTTTTGGGGCCTTTGTAGGCCCCGGGCCTTGTTCGGCCCCATGCACTCCGAATTCCCATTCAGCGGGTTC | R primer ordered as megaprimer to create P123:23 construct for β-gal assay |
| P12F | GAACCCGCTGAATGGGAATTCACAACCGGCGGTAAATACAAATAAACCAAAAGAAAATCTCTGAAAACTATTCGCTGTTTACTGGCCATTAAGTGCAGTCGGCGAAATTGATCAAGCTTTCACACAGGAAACA | F primer ordered as megaprimer to create P12 construct for β-gal assay |
| P12R | TGTTTCCTGTGTGAAAGCTTGATCAATTTCGCCGACTGCACTTAATGGCCAGTAAACAGCGAATAGTTTTCAGAGATTTTCTTTTGGTTTATTTGTATTTACCGCCGGTTGTGAATTCCCATTCAGCGGGTTC | R primer ordered as megaprimer to create P12 construct for β-gal assay |
| P1(2X)F | CGGATCGCACGAACCCGCTGAATGGGAATTCCCAAAAGAAAATCTCTGAAAACTATTTTACTGGCCATTAATCGCTGTTTACTGGCCATTAAGTGCAGTCGGCGAAATTGATCAAGCTTTCACACAGGAAACAGCTATGACCATG | F primer ordered as megaprimer to create P1(2X) construct for β-gal assay |
| P1(2X)R | CATGGTCATAGCTGTTTCCTGTGTGAAAGCTTGATCAATTTCGCCGACTGCACTTAATGGCCAGTAAACAGCGATTAATGGCCAGTAAAATAGTTTTCAGAGATTTTCTTTTGGGAATTCCCATTCAGCGGGTTCGTGCGATCCG | R primer ordered as megaprimer to create P1(2X) construct for β-gal assay |
| P1(3X)F | CGGATCGCACGAACCCGCTGAATGGGAATTCTCTCTGAATTTACTGGCCATTAAAACTATTTTACTGGCCATTAATCGCTGTTTACTGGCCATTAAGTGCAGTCGGCGAAATTGATCAAGCTTTCACACAGGAAACAGCTATGACCATG | F primer ordered as megaprimer to create P1(3X) construct for β-gal assay |
| P1(3X)R | CATGGTCATAGCTGTTTCCTGTGTGAAAGCTTGATCAATTTCGCCGACTGCACTTAATGGCCAGTAAACAGCGATTAATGGCCAGTAAAATAGTTTTAATGGCCAGTAAATTCAGAGAGAATTCCCATTCAGCGGGTTCGTGCGATCCG | R primer ordered as megaprimer to create P1(3X) construct for β-gal assay |
| P12XNF | GAACCCGCTGAATGGGAATTCACAACCGGCGGTTTACTGGCCATTAACCAAAAGAAAATCTCTGAAAACTATTCGCTGTTTACTGGCCATTAAGTGCAGTCGGCGAAATTGATCAAGCTTTCACACAGGAAACA | F primer ordered as megaprimer to create P12XN construct for β-gal assay |
| P12XNR | TGTTTCCTGTGTGAAAGCTTGATCAATTTCGCCGACTGCACTTAATGGCCAGTAAAC AGCGAATAGTTTTCAGAGATTTTCTTTTGGTTAATGGCCAGTAAACCGCCGGTTGTG AATTCCCATTCAGCGGGTTC | R primer ordered as megaprimer to create P12XN construct for β-gal assay |
| P13XNF | GAACCCGCTGAATGGGAATTCGGAGTGCATGGTTTACTGGCCATTAACGGTTTACTGGCCATTAACCAAAAGAAAATCTCTGAAAACTATTCGCTGTTTACTGGCCATTAAGTGCAGTCGGCGAAATTGATCAAGCTTTCACACAGGAAACA | F primer ordered as megaprimer to create P13XN construct for β-gal assay |
| P13XNR | TGTTTCCTGTGTGAAAGCTTGATCAATTTCGCCGACTGCACTTAATGGCCAGTAAAC AGCGAATAGTTTTCAGAGATTTTCTTTTGGTTAATGGCCAGTAAACCGTTAATGGCC AGTAAACCATGCACTCCGAATTCCCATTCAGCGGGTTC | R primer ordered as megaprimer to create P13XN construct for β-gal assay |
| P13X31F | GAACCCGCTGAATGGGAATTCGGAGTGCATGGTTTACTGGCCATTAACGCGGAGTGCATGGCCGGGAACAACCGGCGGTTTACTGGCCATTAACCAAAAGAAAATCTCTGAAAACTATTCGCTGTTTACTGGCCATTAAGTGCAGTCGGCGAAATTGATCAAGCTTTCACACAGGAAACA | F primer ordered as megaprimer to create P13X31 construct for β-gal assay |
| P13X31R | TGTTTCCTGTGTGAAAGCTTGATCAATTTCGCCGACTGCACTTAATGGCCAGTAAAC AGCGAATAGTTTTCAGAGATTTTCTTTTGGTTAATGGCCAGTAAACCGCCGGTTGTTC CCGGCCATGCACTCCGCGTTAATGGCCAGTAAACCATGCACTCCGAATTCCCATTCA GCGGGTTC | R primer ordered as megaprimer to create P13X31 construct for β-gal assay |
| ctrApET28aF | gcaaatgggtcgcggatccgaattcATGCGCGTACTGTTGATCGAG | F primer to amplify *C. crescentus* *ctrA* for cloning into pET28a vector |
| ctrApET28aR | ggtgctcgagtgcggccgcaagctTCAGGCGGCGTTAACCTGCTC | R primer to amplify *C. crescentus ctrA* for cloning into pET28a vector |
| P1234:23upF | attgaagccggctggcgccaCCAGAAACACCAGGCCCAGC | F primer to amplify upstream fragment for creating P*_pilA_* chromosomal mutations |
| P1234:1upR | acggcctggcCAGGGCCCAGCGAATAGTTTTCAGAGATTTTCTTTTGG | R primer to amplify upstream fragment for creating P1234:1 strain |
| P1234:1dnF | ctgggccctgGCCAGGCCGTGCAGTCGGCGAAATTGATC | F primer to amplify downstream fragment for creating P1234:1 strain |
| P1234:23dnF | caaggcccggGGCCTACAAAGGCCCCAAAAGAAAATCTCTGAAAACTATTCGC | F primer to amplify downstream fragment for creating P*_pilA_* chromosomal mutations |
| P1234:23dnR | cgtcacggccgaagctagcgGCCGGTGTACAGCATGAACG | R primer to amplify downstream fragment for creating P*_pilA_* chromosomal mutations |
| P1234:2upR | tttgtaggccCCGTTATTTGTTCTTAACCATGCAC | R primer to amplify upstream fragment for creating P1234:2 strain |
| P1234:3dnF | caaggcccggTAAATACAAATAAACCAAAAGAAAATCTCTGAAAAC | F primer to amplify downstream fragment for creating P1234:3 strain |
| P1234:23upR | tttgtaggccCCGGGCCTTGTTCGGCCCCATGCACTCCGCGATCGCTG | R primer to amplify upstream fragment for creating P1234:23 strain |

**Table S4: List of primers/oligonucleotides used in this study.**

**
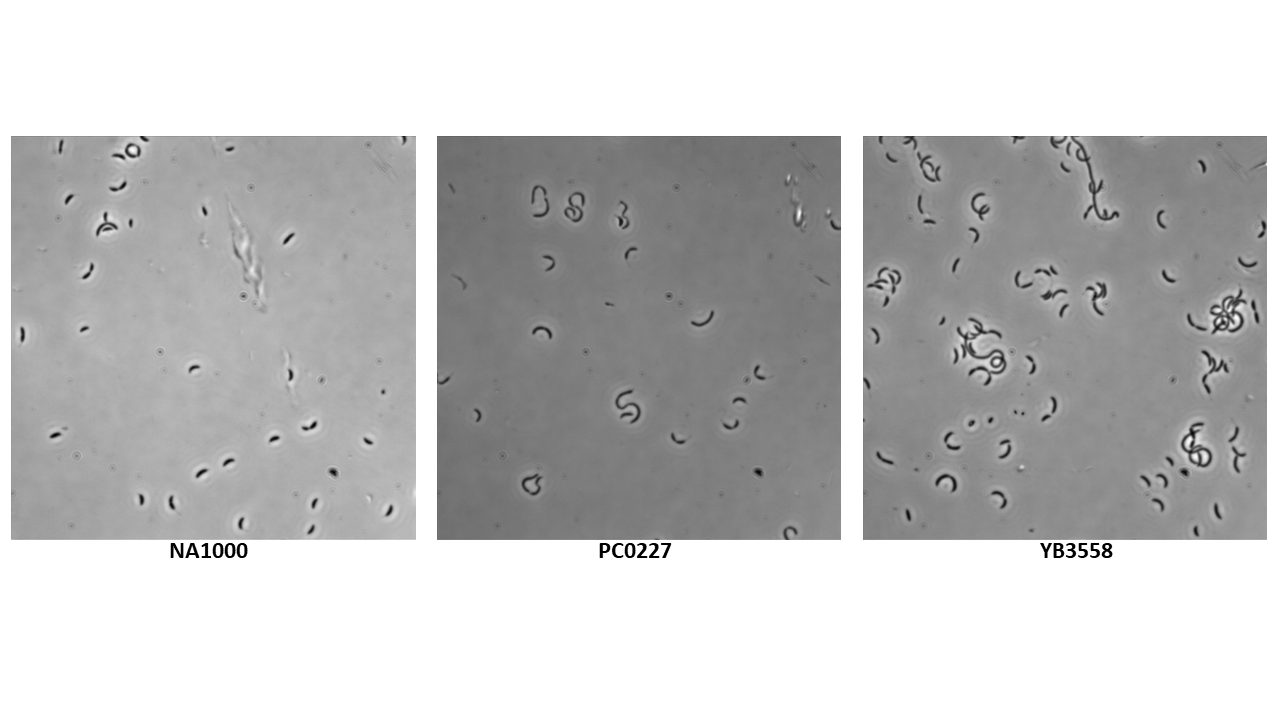
**

**Figure S1: Microscopy images showing the filamentous phenotype of PC0227 and YB3558 compared to WT.**

**
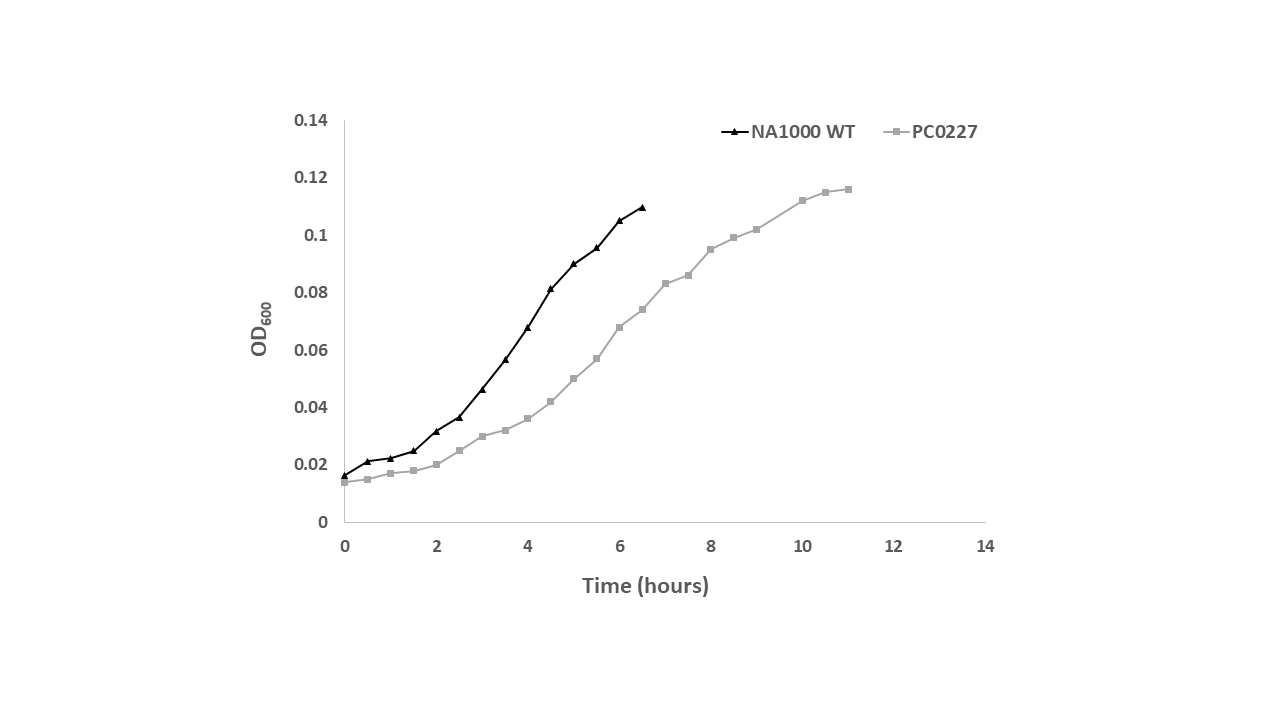
**

**Figure S2: Growth curve for *C. crecentus* NA1000 WT (black traingles) and PC0227 (gray squares) strains. Compared to the WT which has a doubling time of 105 minutes, PC0227 grows at a slower rate with a doubling time of 129 minutes.**

**
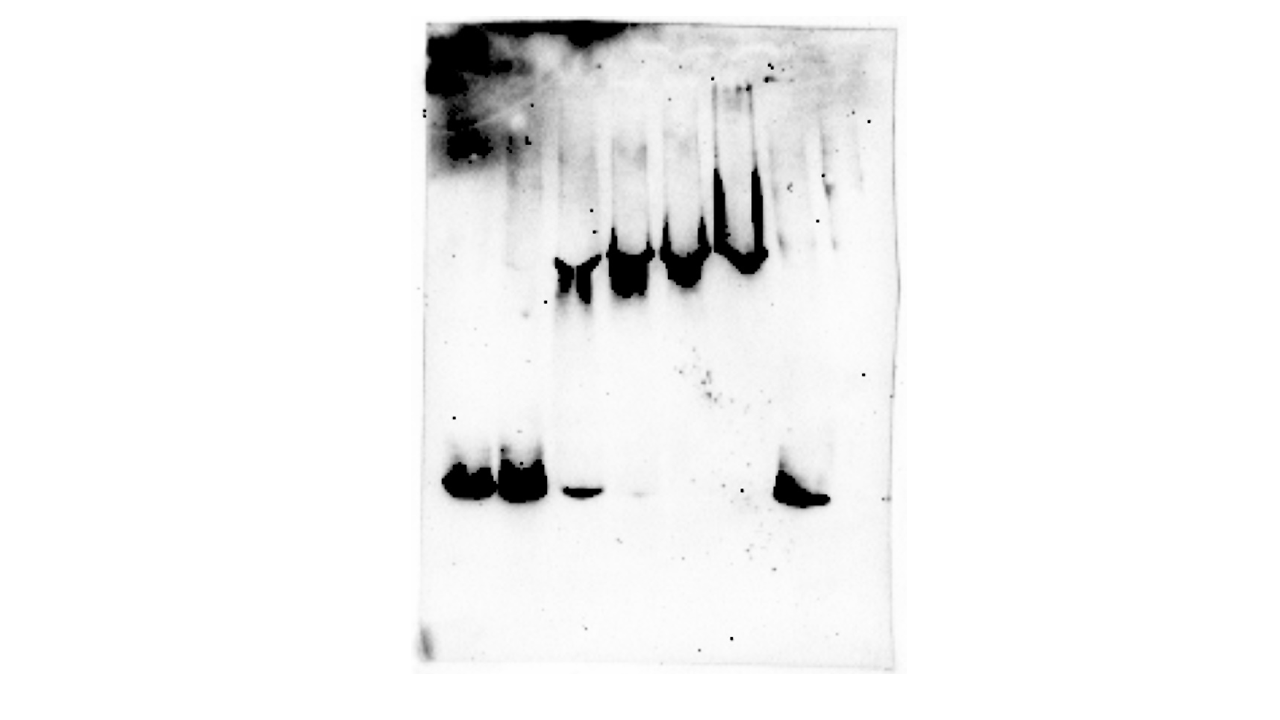
**

**Figure S3: Uncropped EMSA membrane for P123.**

**
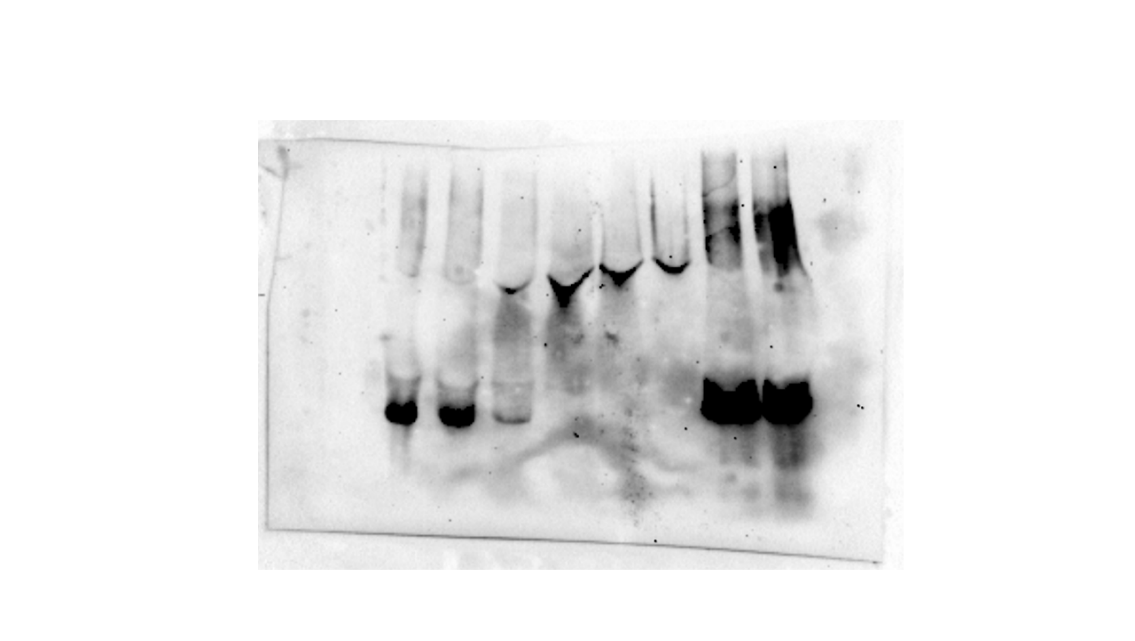
**

**Figure S4: Uncropped EMSA membrane for P123:1.**

**
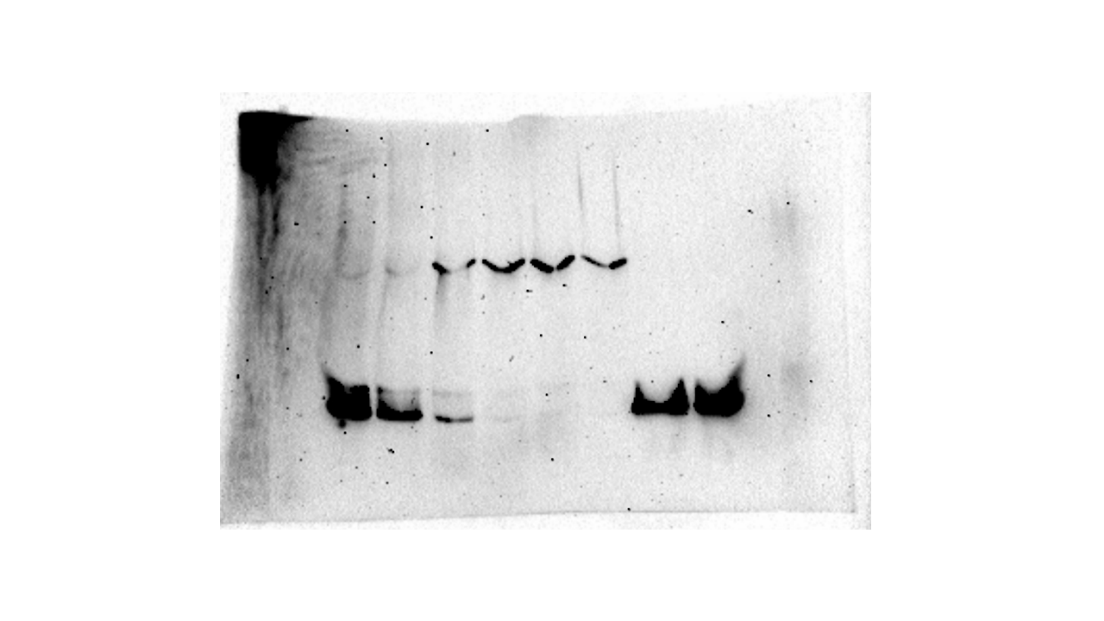
**

**Figure S5: Uncropped EMSA membrane for P123:2.**

**
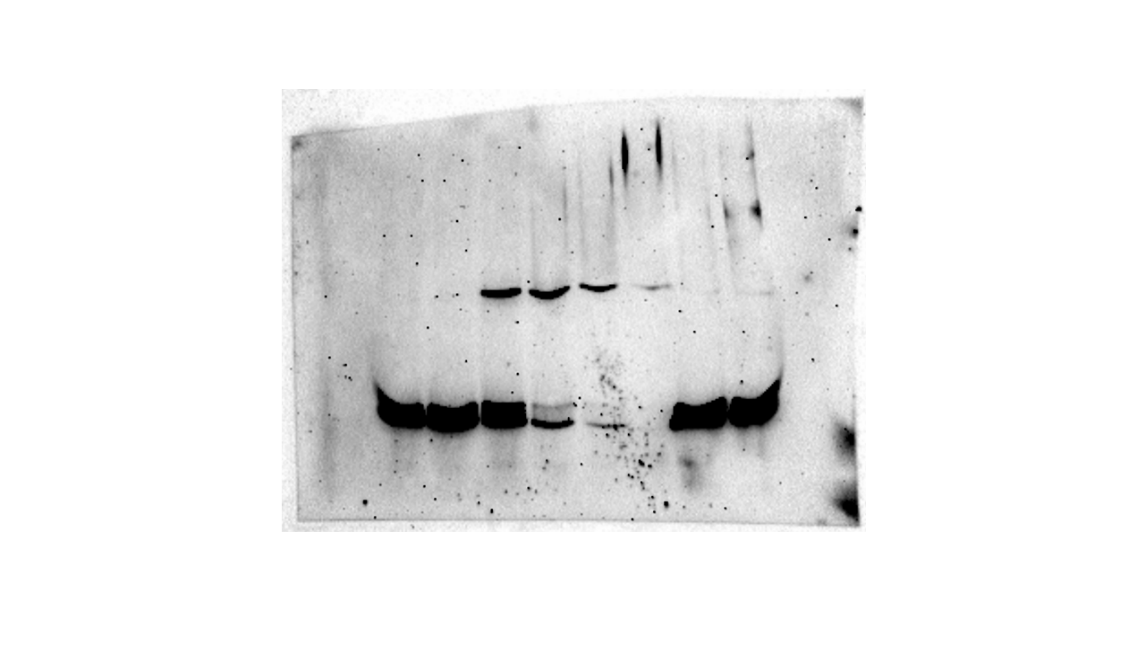
**

**Figure S6: Uncropped EMSA membrane for P123:3.**

**
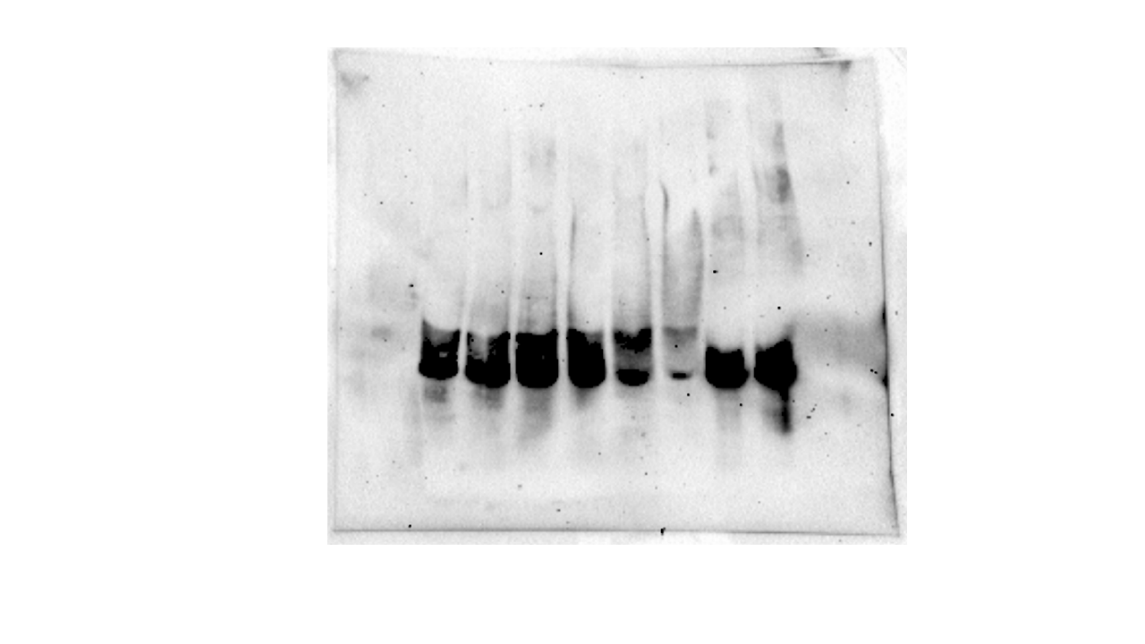
**

**Figure S7: Uncropped EMSA membrane for P123:12.**

**
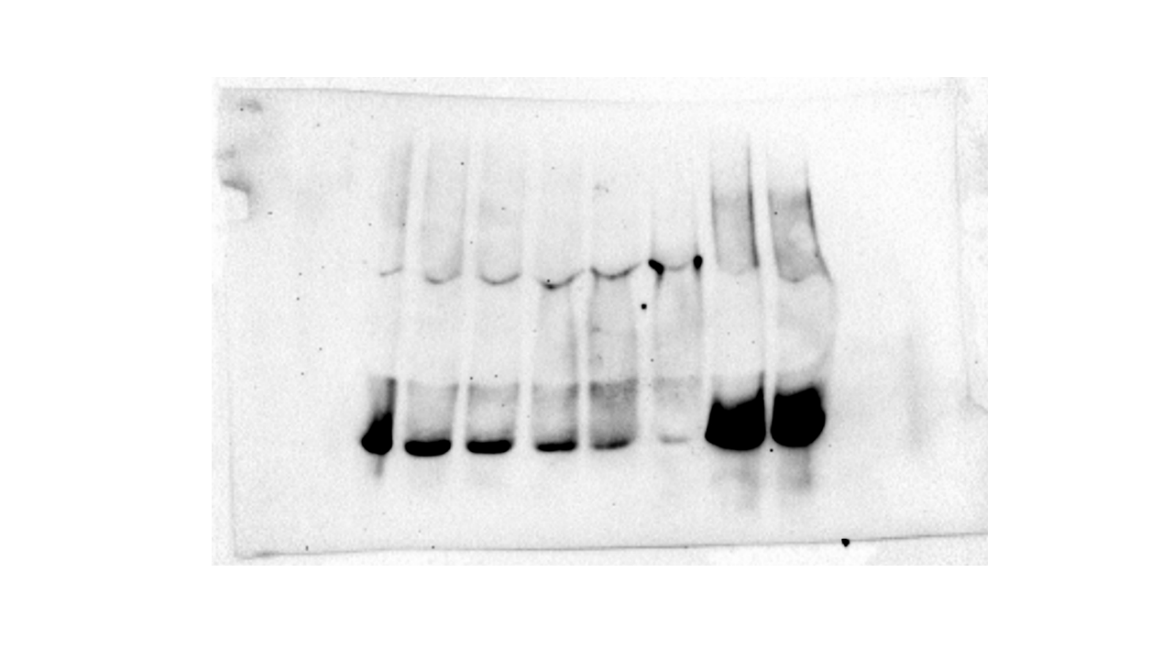
**

**Figure S8: Uncropped EMSA membrane for P123:13.**

**
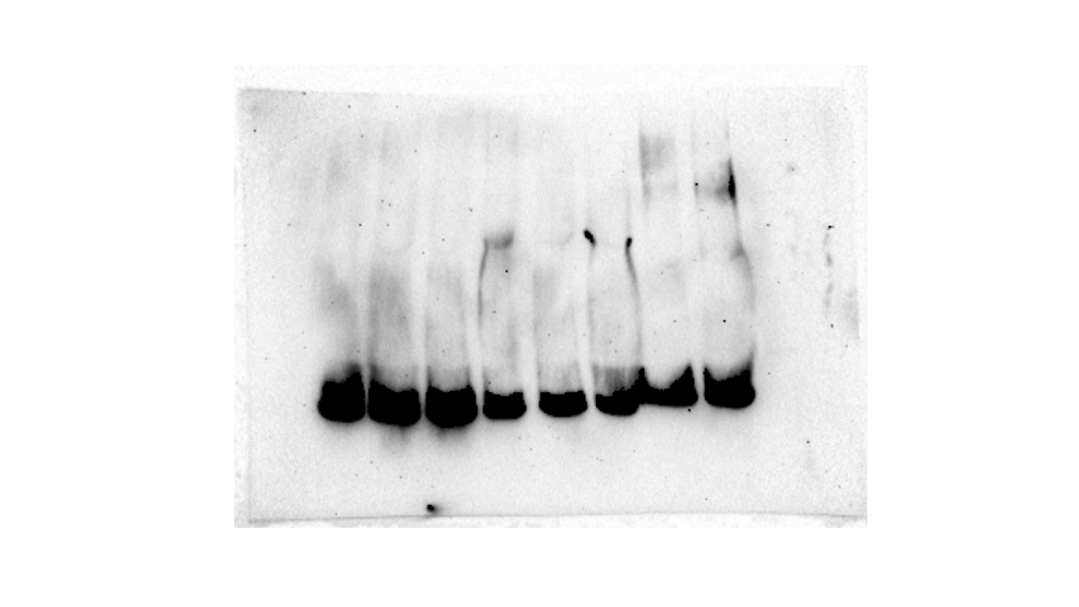
**

**Figure S9: Uncropped EMSA membrane for P1.**

**
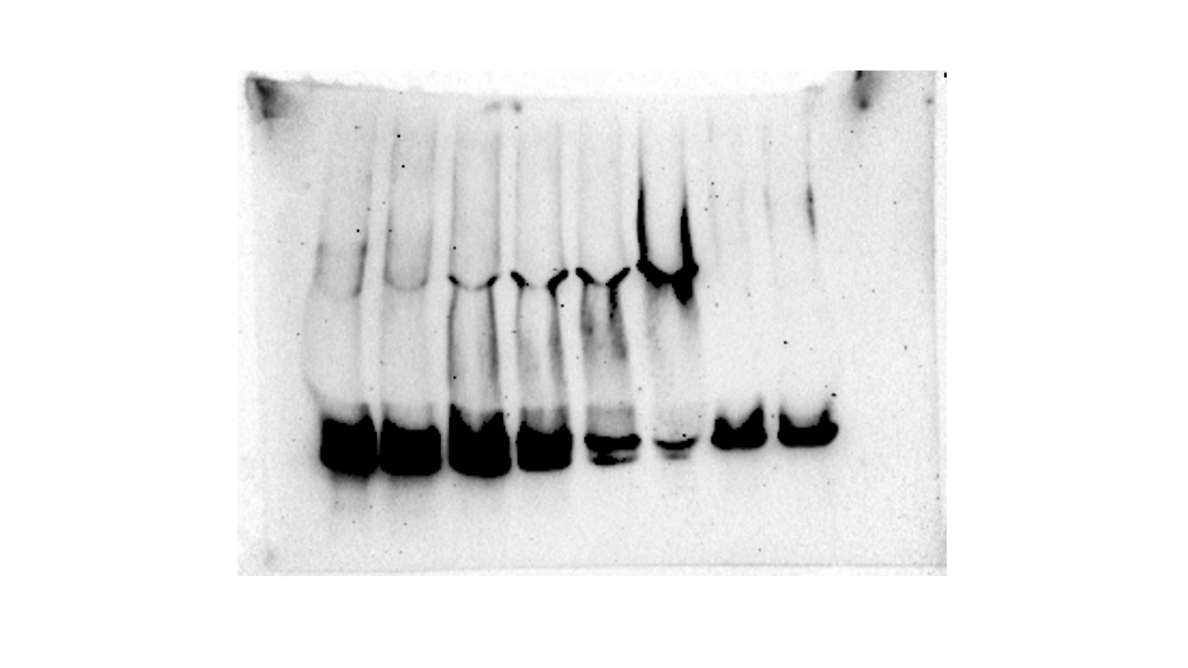
**

**Figure S10: Uncropped EMSA membrane for P1 (2X).**

**
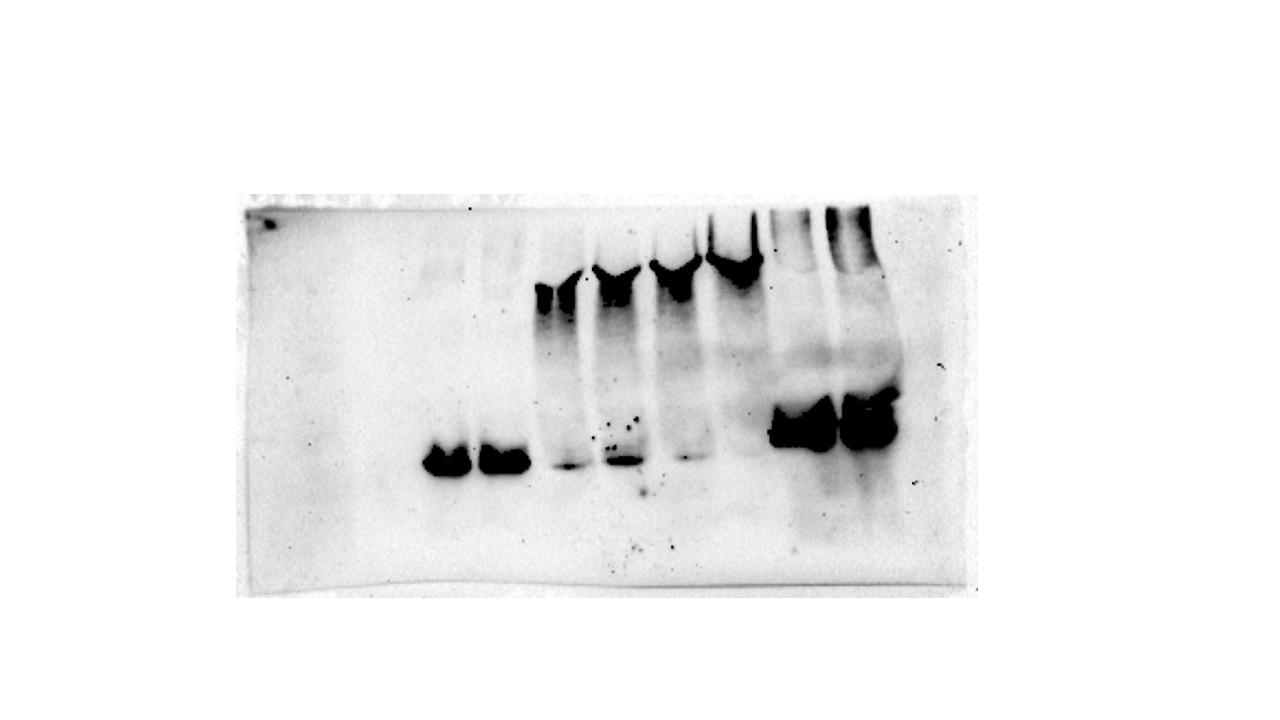
**

**Figure S11: Uncropped EMSA membrane for P1(3X).**

**
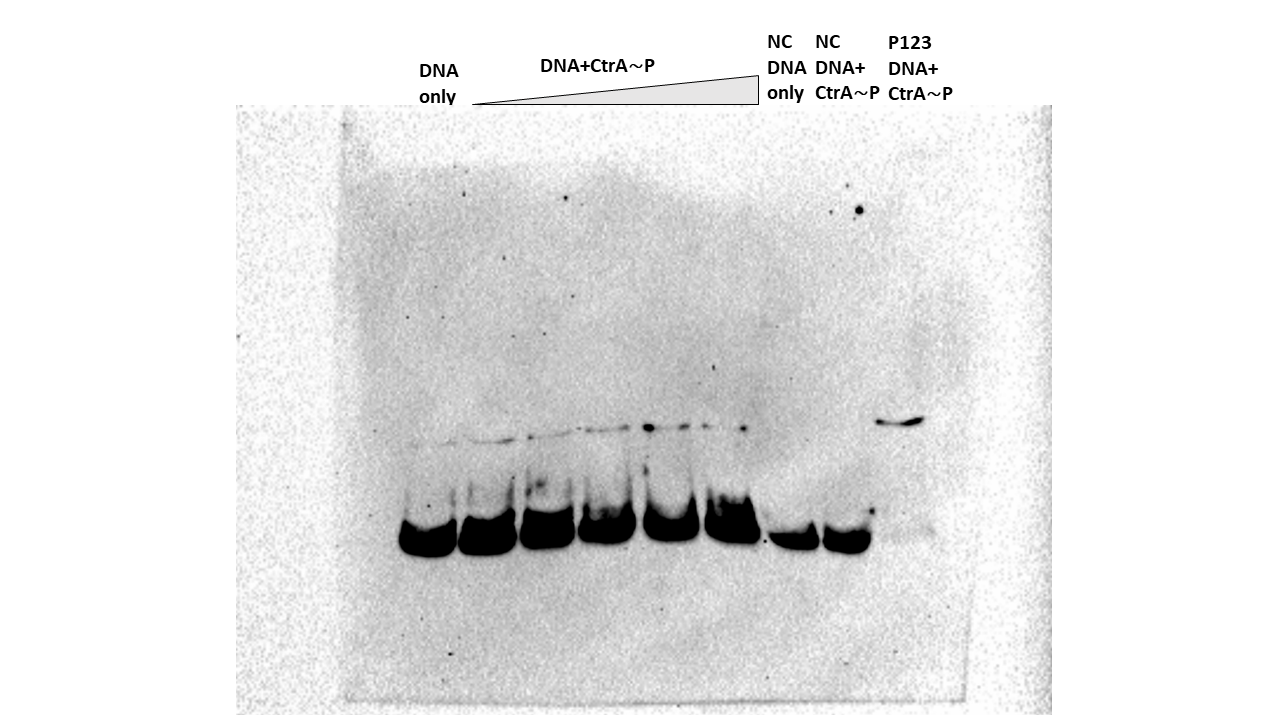
**

**Figure S12: Uncropped EMSA membrane for P123:23 probe that includes the P123 probe with CtrA on the last lane as a positive control for CtrA activity and binding.** All lanes contained 100 ng DNA and 1 ng Poly didC. NC indicates a negative control DNA fragment that has all half-sites in Sites 1, 2 and 3 mutated to GGCC and Site 4 is absent. The first lane had DNA only and the second to sixth lanes had DNA and 0.17 µM, 0.33 µM, 0.5 µM, 0.66 µM and 1.65 µM of phosphorylated CtrA respectively. The seventh and eighth lanes were negative controls with NC DNA only and NC DNA with 1.65 µM of phosphorylated CtrA respectively. P123 DNA with 1.65 µM CtrA was included on the last lane as a positive control.


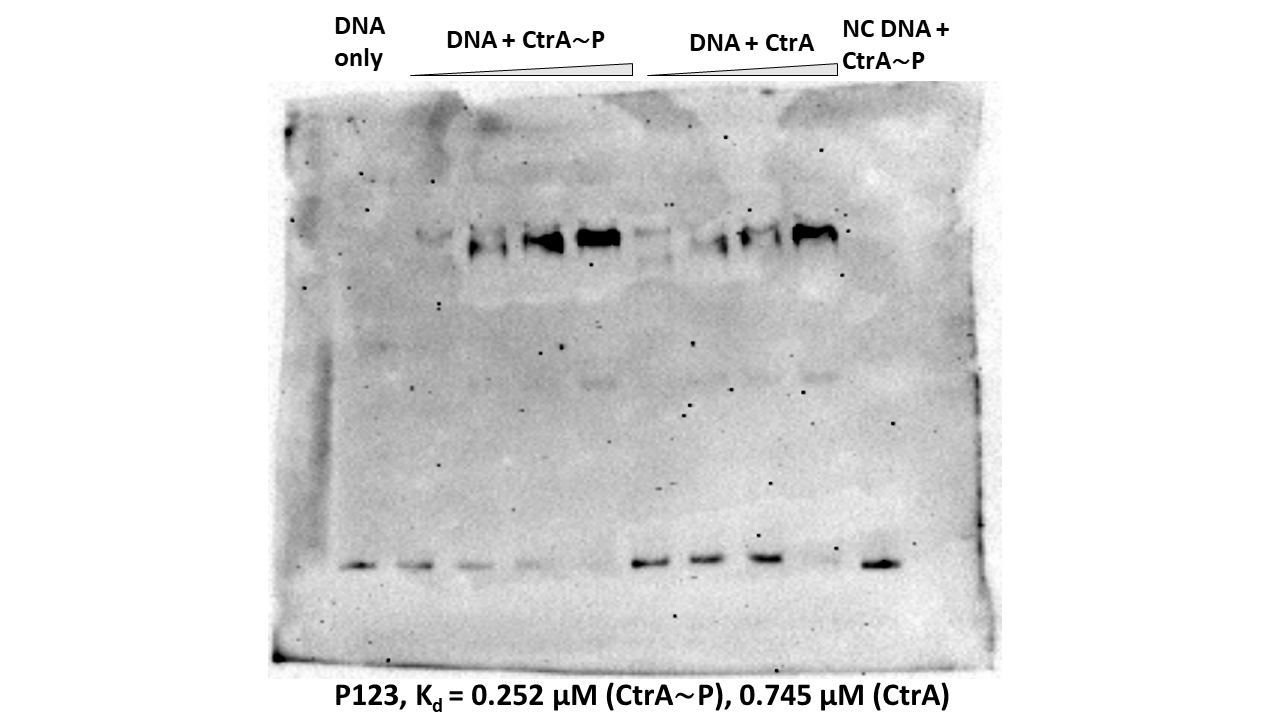


**Figure S13: Uncropped EMSA membrane for P123 with and without phosphorylation.** All lanes contained 100 ng DNA and 1 ng Poly didC. NC indicates a negative control DNA fragment that has all half-sites in Sites 1, 2 and 3 mutated to GGCC and Site 4 is absent. The first lane had DNA only and the second to fifth lanes had DNA and 0.14 µM, 0.28 µM, 0.56 µM and 1.12 µM of phosphorylated CtrA respectively. The sixth to ninth lanes had DNA and 0.14 µM, 0.28 µM, 0.56 µM and 1.12 µM of unphosphorylated CtrA respectively. The last lane had NC DNA with 1.12 µM of phosphorylated CtrA.


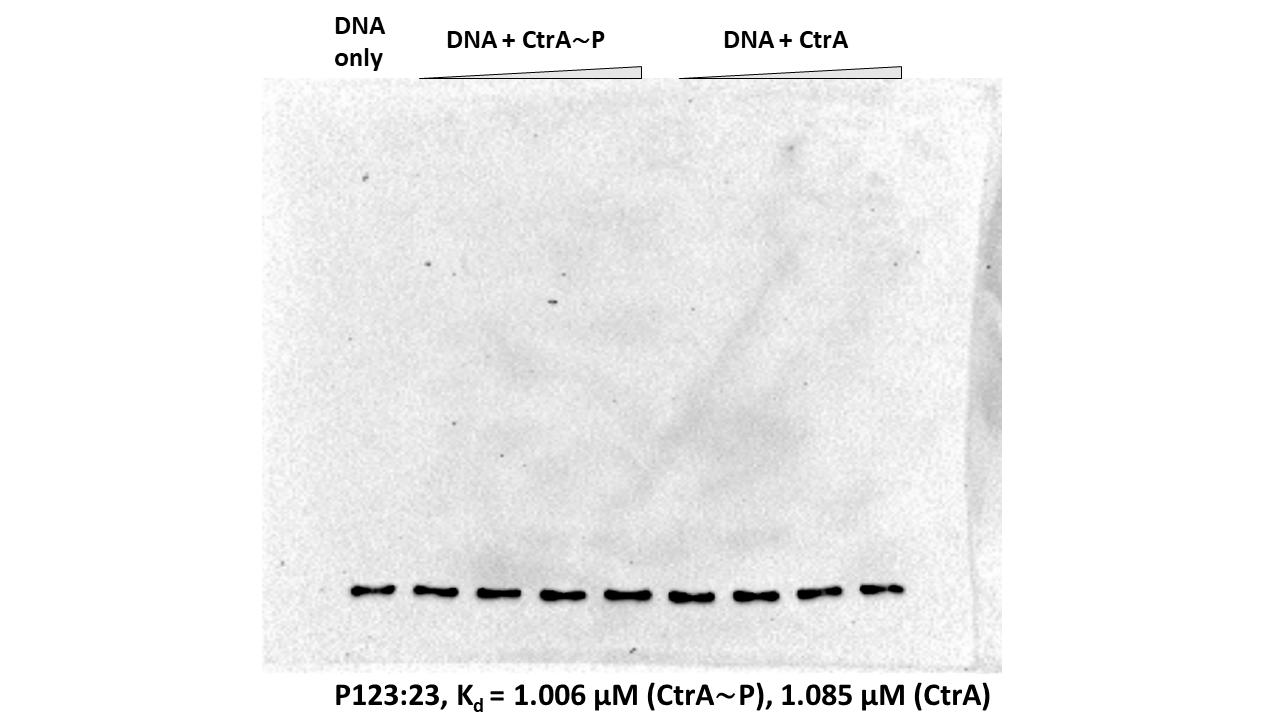


**Figure S14: Uncropped EMSA membrane for P123:23 with and without phosphorylation**. All lanes contained 100 ng DNA and 1 ng Poly didC. NC indicates a negative control DNA fragment that has all half-sites in Sites 1, 2 and 3 mutated to GGCC and Site 4 is absent. The first lane had DNA only and the second to fifth lanes had DNA and 0.14 µM, 0.28 µM, 0.56 µM and 1.12 µM of phosphorylated CtrA respectively. The sixth to ninth lanes had DNA and 0.14 µM, 0.28 µM, 0.56 µM and 1.12 µM of unphosphorylated CtrA respectively.

**
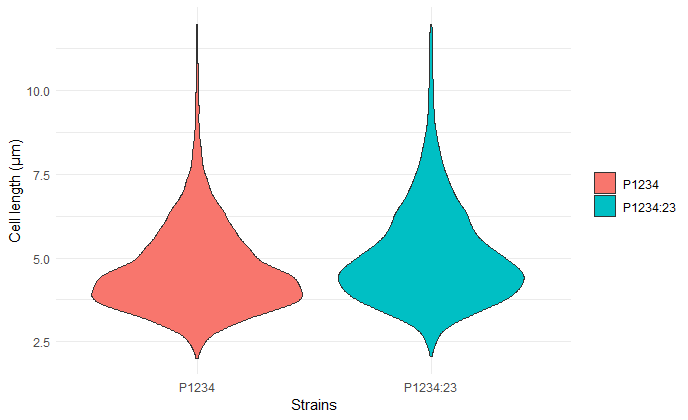
**

**Figure S15: A violin plot showing the difference in size (length) distribution of WT (P1234) and P1234:23 cells captured from the piliated cell bead-binding experiment.**

**
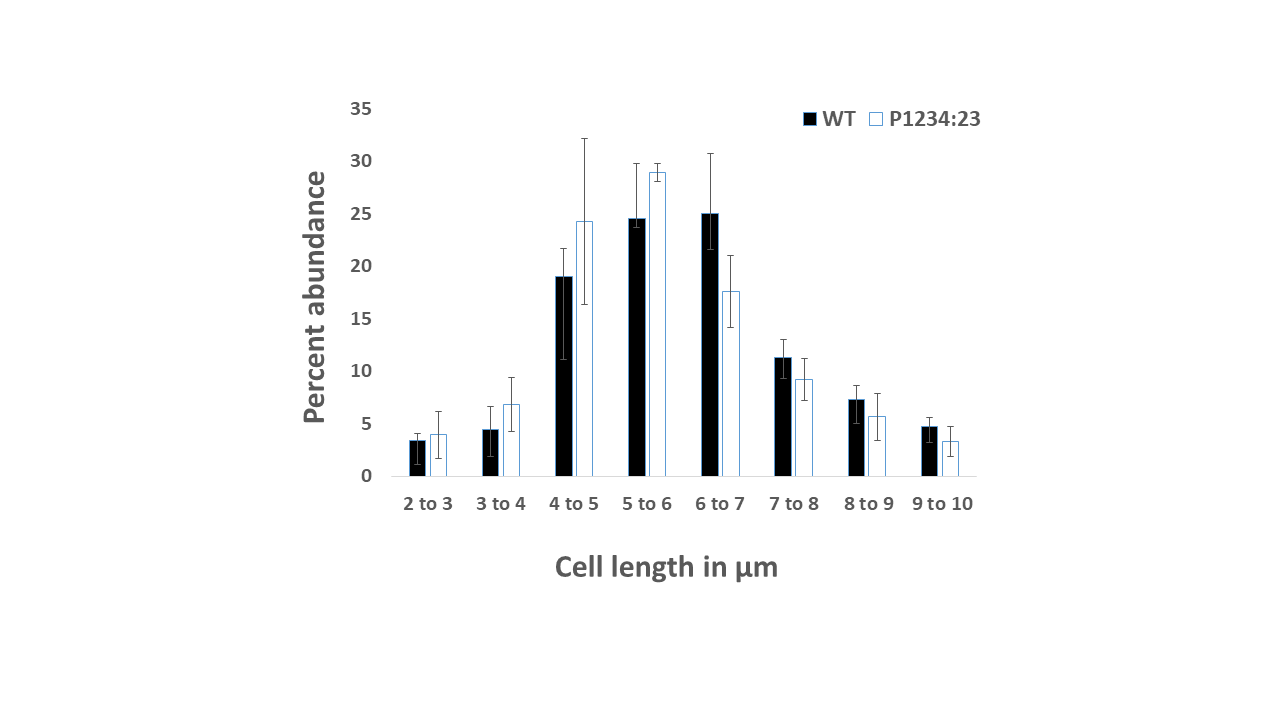
**

**Figure S16: A bar-graph showing the size (length) distribution of WT (P1234) and P1234:23 cells without bead enrichment.**

**
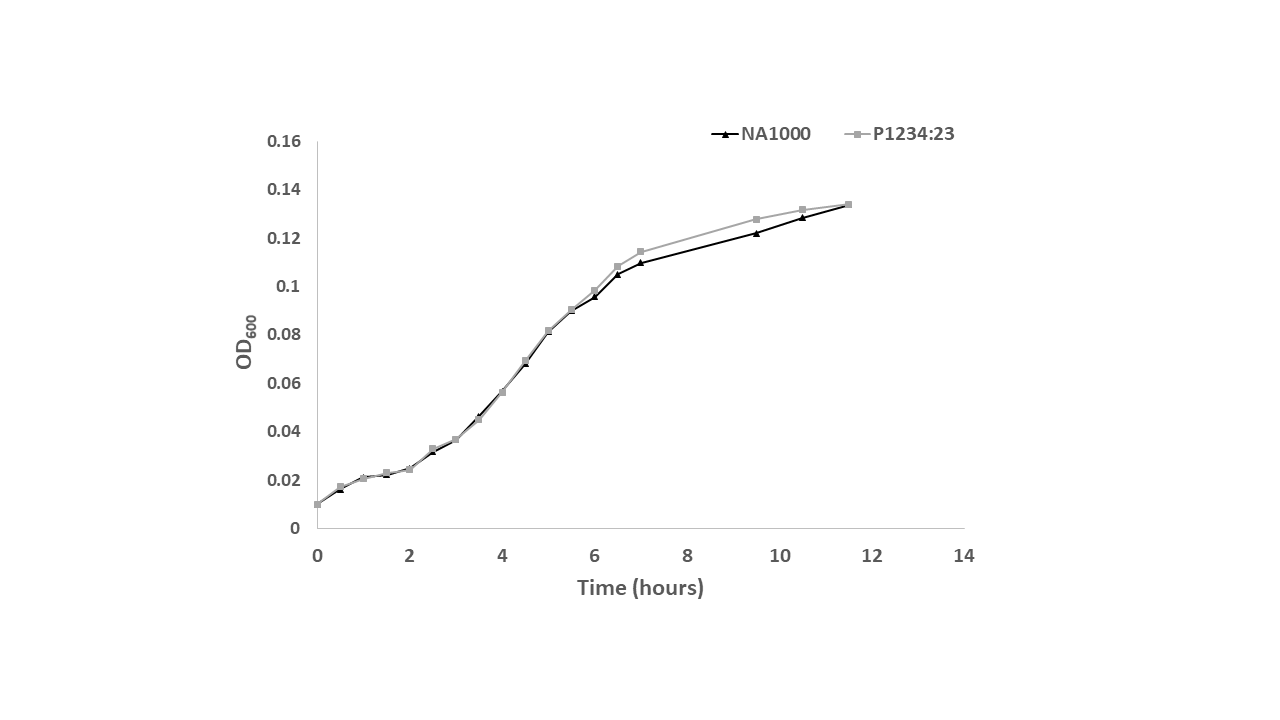
**

**Figure S17: Growth curve for *C. crecentus* NA1000 WT (black traingles) and P1234:23 (gray squares) strains. Both strains have the same growth rate with a doubling time of 105 minutes.**


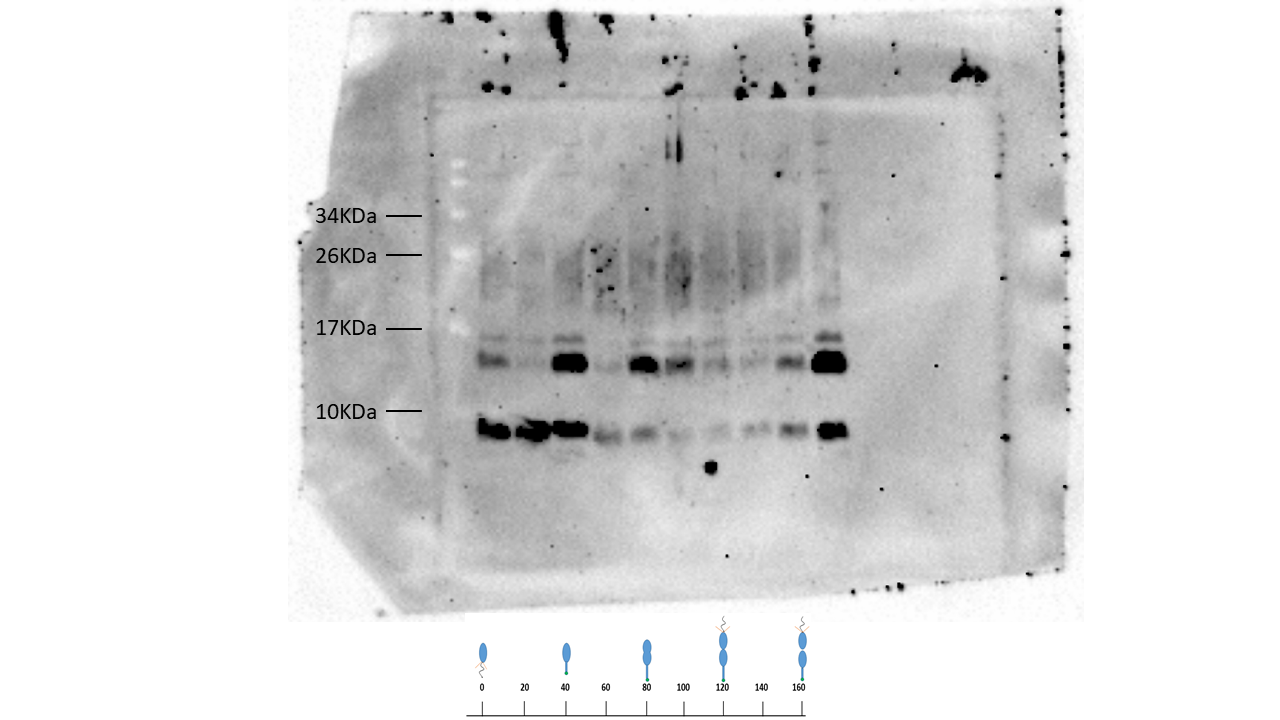


**Figure S18: Uncropped Western Blot with polyclonal anti-PilA antibodies** (5–8) **for synchronized cultures of P1234 (WT) showing PilA (**∼**9kDa) accumulation over different timepoints during the cell cycle at 20 minute-intervals starting with timepoint 0.**


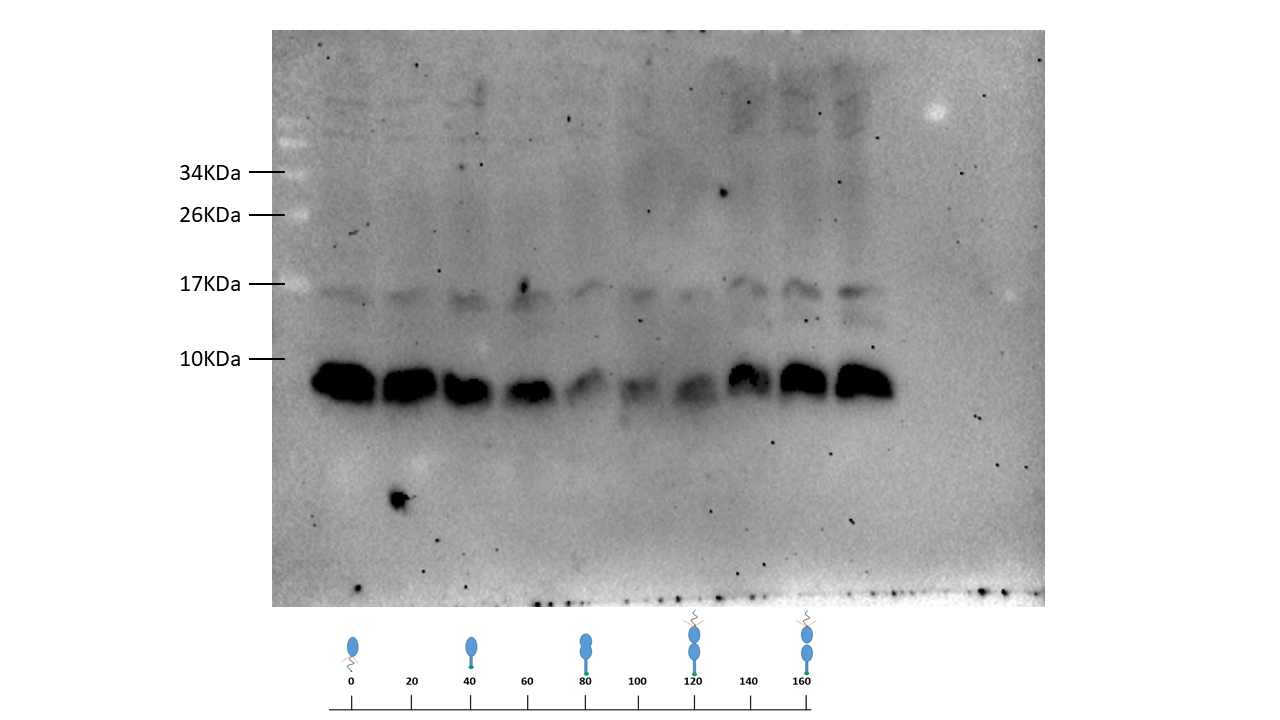


**Figure S19: Uncropped Western Blot with polyclonal anti-PilA antibodies for synchronized cultures of P1234:2 showing PilA accumulation over different timepoints during the cell cycle at 20 minute-intervals starting with timepoint 0. The PilA band is seen below the 10kDa marker.**

**
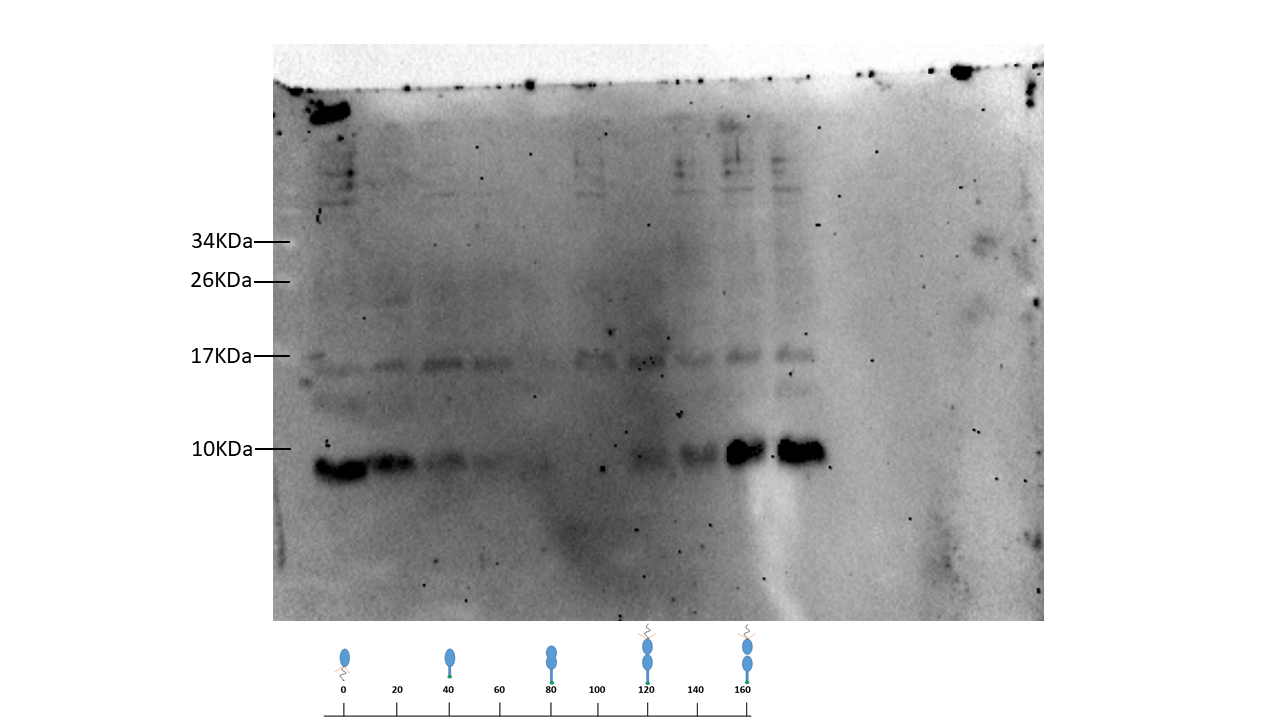
**

**Figure S20: Uncropped Western Blot with polyclonal anti-PilA antibodies for synchronized cultures of P1234:3 showing PilA accumulation over different timepoints during the cell cycle at 20 minute-intervals starting with timepoint 0. The PilA band is seen below the 10kDa marker.**

**
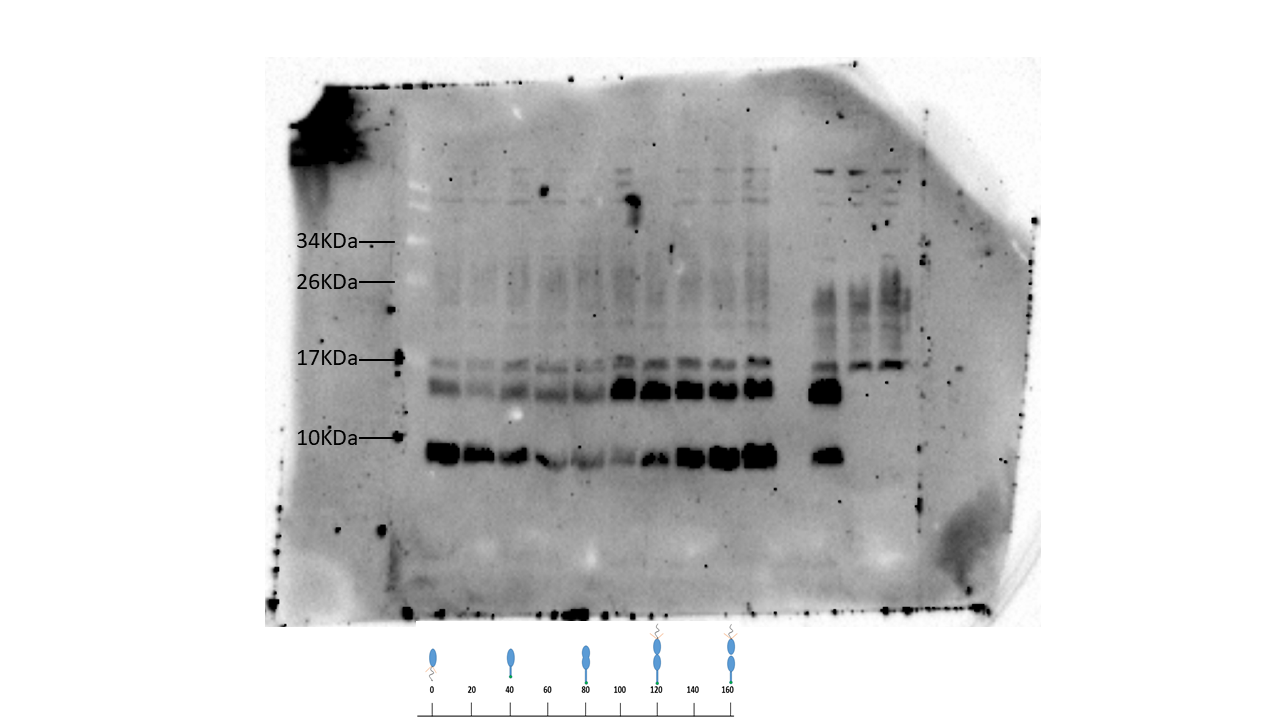
**

**Figure S21: Uncropped Western Blot with polyclonal anti-PilA antibodies for synchronized cultures of P1234:3 showing PilA accumulation over different timepoints during the cell cycle at 20 minute-intervals starting with timepoint 0. The PilA band is seen below the 10kDa marker. The last three lanes to the right show bands for whole cell lysates of overnight cultures of P1234 (positive control) and 2 replicates of Δ*pilA* strain (negative control), respectively.**

**References**

1. Kelly AJ, Sackett MJ, Din N, Quardokus E, Brun Y V. Cell cycle-dependent transcriptional and proteolytic regulation of FtsZ in Caulobacter. Genes Dev. 1998;12(6):880–93.

2. Evinger M, Agabian N. Envelope associated nucleoid from Caulobacter crescentus stalked and swarmer cells. J Bacteriol. 1977;132(1):294–301.

3. Ellison CK, Kan J, Dillard RS, Kysela DT, Ducret A, Berne C, et al. Obstruction of pilus retraction stimulates bacterial surface sensing. Science (80- ). 2017;358(6362):535–8.

4. Curtis PD, Klein D, Brun Y V. Effect of a ctrA promoter mutation, causing a reduction in CtrA abundance, on the cell cycle and development of Caulobacter crescentus. BMC Microbiol [Internet]. 2013;13(1):1. Available from: BMC Microbiology

5. Skerker JM, Shapiro L. Identification and cell cycle control of a novel pilus system in Caulobacter crescentus. EMBO J. 2000;19(13):3223–34.

6. Viollier PH, Sternheim N, Shapiro L. A dynamically localized histidine kinase controls the asymmetric distribution of polar pili proteins. EMBO J. 2002;21(17):4420–8.

7. Curtis PD, Quardokus EM, Lawler ML, Guo X, Klein D, Chen JC, et al. The scaffolding and signalling functions of a localization factor impact polar development. Mol Microbiol [Internet]. 2012 May;84(4):712–35. Available from: http://www.ncbi.nlm.nih.gov/pubmed/22512778

8. Matteo Sangermani, Isabelle Hug, Nora Sauter, Thomas Pfohl UJ. crossm. Am Soc Microbiol. 2019;10(3).
